# Crystal structures of three 3,4,5-tri­meth­oxy­benzamide-based derivatives

**DOI:** 10.1107/S2056989016005958

**Published:** 2016-04-15

**Authors:** Ligia R. Gomes, John Nicolson Low, Catarina Oliveira, Fernando Cagide, Fernanda Borges

**Affiliations:** a REQUIMTE, Departamento de Química e Bioquímica, Faculdade de Ciências da Universidade do Porto, Rua do Campo Alegre, 687, P-4169-007, Porto, Portugal; b FP-ENAS-Faculdade de Ciências de Saúde, Escola Superior de Saúde da UFP, Universidade Fernando Pessoa, Rua Carlos da Maia, 296, P-4200-150 Porto, Portugal; c Department of Chemistry, University of Aberdeen, Meston Walk, Old Aberdeen AB24 3UE, Scotland; d CIQ/Departamento de Quιmica e Bioquιmica, Faculdade de Ciências, Universidade do Porto, 4169-007 Porto, Portugal

**Keywords:** crystal structure, benzamide, hydrogen bonding

## Abstract

The crystal structures of three benzamide derivatives, *viz. N*-(6-hy­droxy­hex­yl)-3,4,5-tri­meth­oxy­benzamide, C_16_H_25_NO_5_, (**1**), *N*-(6-anilinohex­yl)-3,4,5-tri­meth­oxy­benzamide, C_22_H_30_N_2_O_4_, (**2**), and *N*-(6,6-di­eth­oxy­hex­yl)-3,4,5-tri­meth­oxy­benzamide, C_20_H_33_NO_6_, (**3**), are described. These compounds differ only in the substituent at the end of the hexyl chain and the nature of these substituents determines the differences in hydrogen bonding between the mol­ecules. In each mol­ecule, the *m*-meth­oxy substituents are virtually coplanar with the benzyl ring, while the *p*-meth­oxy substituent is almost perpendicular. The carbonyl O atom of the amide rotamer is *trans* related with the amidic H atom. In each structure, the benzamide N—H donor group and O acceptor atoms link the mol­ecules into *C*(4) chains. In **1**, a terminal –OH group links the mol­ecules into a *C*(3) chain and the combined effect of the *C*(4) and *C*(3) chains is a ribbon made up of screw related *R*_2_^2^(17) rings in which the ⋯O—H⋯ chain lies in the centre of the ribbon and the tri­meth­oxy­benzyl groups forms the edges. In **2**, the combination of the benzamide *C*(4) chain and the hydrogen bond formed by the terminal N—H group to an O atom of the 4-meth­oxy group link the mol­ecules into a chain of *R*_2_^2^(17) rings. In **3**, the mol­ecules are linked only by *C*(4) chains.

## Abstract 2

These benzamide derivatives differ only in the substituent that terminates the hexyl chain and the nature of these substituents determines the differences in hydrogen bonding between the mol­ecules.

## Chemical context   

Phenolic acids are widely distributed in the plant kingdom and exist in significant qu­anti­ties in the human diet (*e.g*. in fruits and vegetables). Like other phenolic compounds they are recognized for their health benefits, which are linked to their biological properties, particularly anti-oxidant, anti-inflammatory and anti­cancer properties (Benfeito *et al.*, 2013 ▸, Roleira *et al.*, 2015 ▸, Garrido *et al.*, 2013 ▸, Teixeira *et al.*, 2013 ▸). Within this framework, our project has been focused on the synthesis of new mol­ecules based on the benzoic acid scaffold. Accordingly, herein we describe the syntheses and structures of three new benzamide derivatives, *viz. N*-(6-hy­droxy­hex­yl)-3,4,5-tri­meth­oxy­benzamide (**1**) *N*-(6-anilinohex­yl)-3,4,5-tri­meth­oxy­benzamide (**2**) and *N*-(6,6-di­eth­oxy­hex­yl)-3,4,5-tri­meth­oxy­benzamide (**3**).

## Structural commentary   

**Figure 1:**
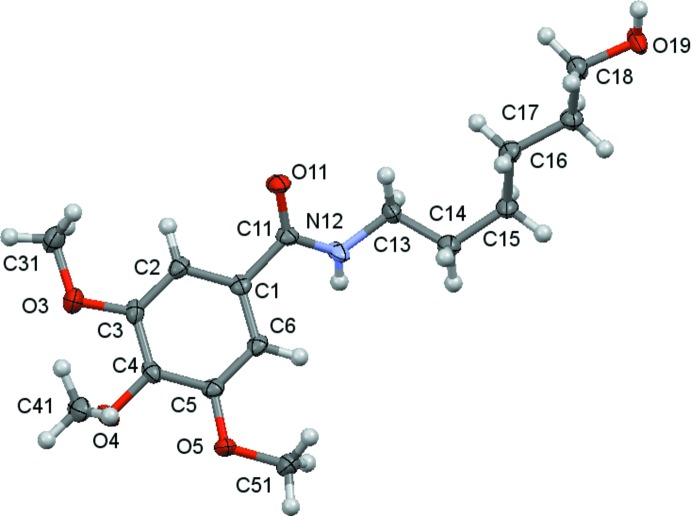
A view of the asymmetric unit of (**1**) with the atom-numbering scheme. Displacement ellipsoids are drawn at the 70% probability level.

The mol­ecular structures of compounds **1**, **2** and **3** are shown in Figs. 1 ▸–3 ▸ ▸. The mol­ecules consist of a tri­meth­oxy­benzamide ‘head’ that is linked to a six-carbon-atom alkyl chain ‘tail’ that ends with different functional groups: a hydroxyl group for **1**, a phenyl­amino group for **2** and a dieth­oxy group for **3**. In spite of having the same ‘head’ and ‘tail’, the differences observed for their mol­ecular conformations are not only due to the different ‘end tail’ functional groups. Thus, the analysis of the mol­ecular conformations will be performed on a comparative basis encompassing the following: (i) the relative positions of the meth­oxy substituents on the aromatic ring; (ii) the conformation of the amide unit and (iii) the conformation of the alkyl chain. The specifics of the substituents at the end of the alkyl chain determine the differences in the supra­molecular structures, as discussed in the next section.
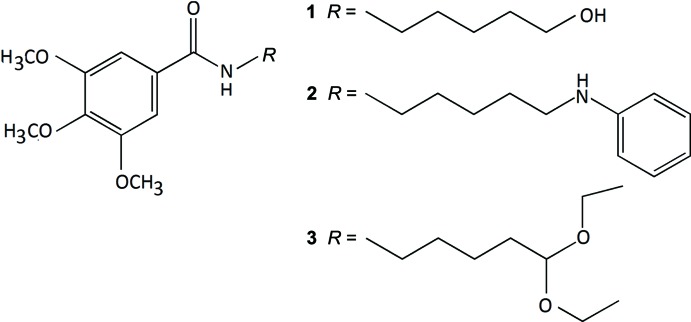


The *m*-meth­oxy substituents are virtually co-planar with the benzene ring and are *trans* related with respect to the *p*-carbon atom of the ring [the maximum deviation of the meth­oxy carbon atom to the best plane of the phenyl ring is 0.238 (1) Å in **2**], while the *p*-meth­oxy group is nearly perpendicular [the minimum deviation of the meth­oxy carbon atom to the best plane of the benzene ring being 0.923 (2) Å, also in **2**]. These relative positions agree with previous predictions of theoret­ical calculations for the stabilization energies for meth­oxy-group conformations attached to aromatic rings (Tsuzuki *et al.*, 2002 ▸), which suggested that, while co-planarity is the most stable conformation when there is only one meth­oxy substit­uent on the aromatic ring, the perpendicular conformation may appear as an alternative one when two *vicinal* meth­oxy groups are present. According to these authors, this spatial arrangement is stabilized by a short C—H⋯O contact between the neighbouring groups. As can be seen in Tables 4 ▸, 5 ▸ and 6 ▸, the shortest distances between a methyl H atom and a *vicinal* meth­oxy O atom are 2.44, 2.33 and 2.37 Å for **1**, **2** and **3**, respectively, which do suggest the possibility of a very weak inter­action.

In the amide rotamer, the carbonyl oxygen atom is in a *trans* position with respect to the hydrogen atom of the amidic nitro­gen atom for all compounds, and so, the trimeth­oxy phenyl group is also *trans* related to the alkyl chain. The rotation of the trimeth­oxy phenyl substituent with respect to the amide rotamer around the C11—C1 bond may be evaluated by the N12—C11—C1—C6 torsion angle, whose values are given in Tables 1 ▸–3 ▸ ▸. The mean planes between the C1 benzene ring and the mean plane of the three atoms O11, C11 and N12 are 35.1 (3), 12.00 (16) and 20.19 (14)°, respectively, for **1**, **2** and **3**, showing that the substituent in **2** is significantly less distorted than in the others. In **1** and in **2**, the sense of rotation is anti­clockwise.

The freedom of rotation around the N—C(alk­yl) bond together with the regular tetra­hedral geometry of the *sp* ^3^-hybridized carbon atoms allows the mol­ecules to acquire very different conformational profiles for the alkyl chain as is observed in the C11—N12—C13—C14 torsion angles [129.1 (3) for **1**, −112.80 (13) for **2** and 114.65 (12)° for **3**], as well as the direction of the alkyl chain with respect to the N12—C13 bond, which primarily affects the relative position of the alkyl ‘tail’ with respect to the benzamide moiety. Considering the disposition of the amide rotamer: in **1** and in **3** the alkyl chain is directed backwards from the amide plane and in **2** forward from that plane. This affects the general shape of the mol­ecules, as can be better visualized in Figs. 7 ▸–9 ▸ ▸. So, in spite of the consistent zigzag shape of the remaining alkyl chain those mol­ecules have entirely different spatial arrangements.

## Supra­molecular features   

### Hydrogen Bonding and short contacts   

**Figure 4:**
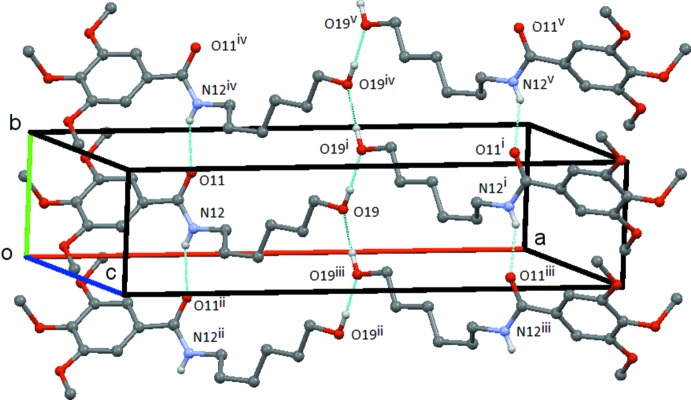
Compound **1**: view of the ribbon structure formed by the N12—H12⋯O11 and O19—H19⋯O19 hydrogen bonds. Hydrogen atoms not involved in the hydrogen bonding are omitted. Symmetry codes: (i) −*x* + 1, −*y* + 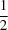, −*z* + 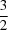; (ii) −*x*, −*y* − 1, −*z* + 1; (iii) −*x* + 1, −*y* − 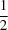, −*z* + 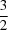; (iv) −*x* + 1, −*y* + 1, −*z* + 1; (v) −*x* + 1, −*y* + 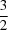, −*z* + 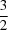.

**Figure 5:**
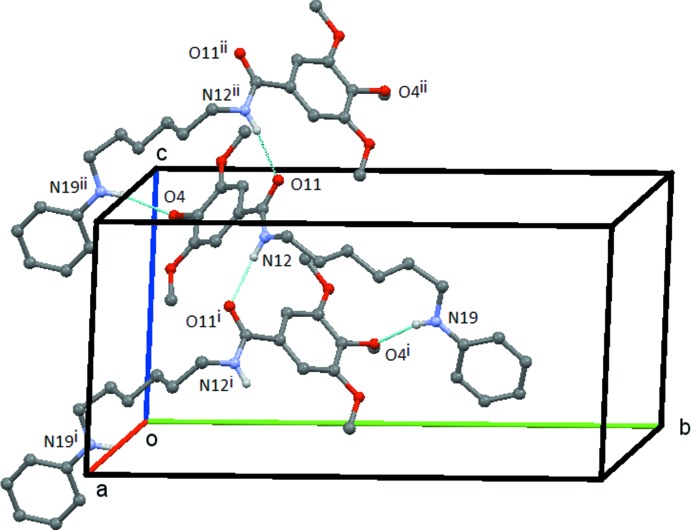
Compound **2**: the chain of rings formed by the inter­action of the N12—H12⋯O11 and N19—H19⋯O4 hydrogen bonds. This chain extends along the *c* axis and is generated by the *c-*glideplane at *y* = 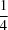. Hydrogen atoms not involved in the hydrogen bonding are omitted. Symmetry codes: (i) *x*, −*y* − 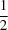, *z* − 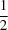; (ii) *x*, −*y* + 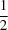, *z* + 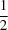.

**Figure 6:**
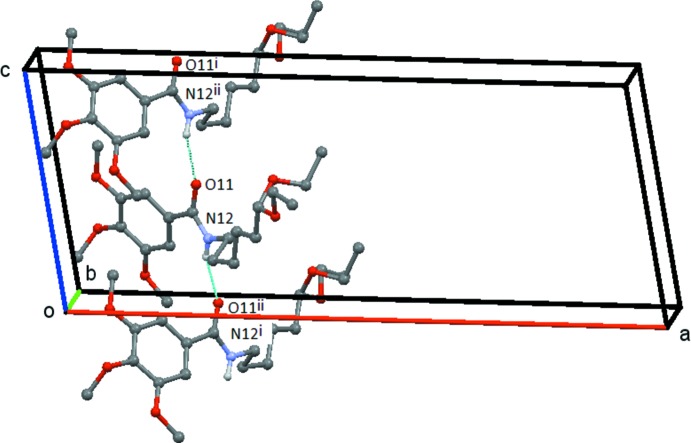
Compound **2**: the simple *C*(4) chain formed by the N12—H12⋯O11 hydrogen bond. This chain extends along the *c* axis and is generated by the *c* glideplane at *y* = 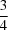. Hydrogen atoms not involved in the hydrogen bonding are omitted. Symmetry codes: (i) *x*, −*y* − 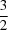, *z* − 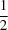; (ii) *x*, −*y* − 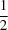, *z* + 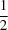.

Tables 4 ▸, 5 ▸ and 6 ▸ show the hydrogen-bonding details for **1**, **2** and **3**, respectively. In each compound, the amide group forms the common *C*(4) chain motif by an N—H⋯O hydrogen bond. In **1**, the N12—-H12⋯O11 chain runs parallel to the *b* axis and adjacent mol­ecules are at unit translation along this axis. The O19—-H19⋯O19 hydrogen bond links the mol­ecules into a *C*(3) chain formed by the action of the twofold screw axis at (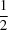, *y*, 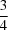). These two chains link the mol­ecules to form a ribbon made up of screw-related 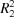
(17) rings, which runs parallel to the *b* axis with the ⋯O—H⋯ chain running up the centre of the ribbon and the tri­meth­oxy­benzyl groups forming the edges (Fig. 4 ▸). In **2**, both the N12—H12⋯O11 and N19—H19⋯O4 hydrogen bonds link the mol­ecules into a chain of 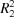
(17) rings, which are bridged by the C11—N12 bond. This chain runs parallel to the *c* axis and is formed by the action of the c-glide plane at 1/4 along the *b* axis (Fig. 5 ▸). In **3**, the N12—H12⋯O11 hydrogen bond links the mol­ecules into a *C*(4) chain, which runs parallel to the *c* axis and which is formed by the action of the *c*-glide plane at 3/4 along the *b* axis, Fig. 6 ▸. Possible weak C—H⋯O inter­actions are detailed in the relevant Tables 4 ▸–6 ▸ ▸.

### Hirshfeld Surfaces   

**Figure 7:**
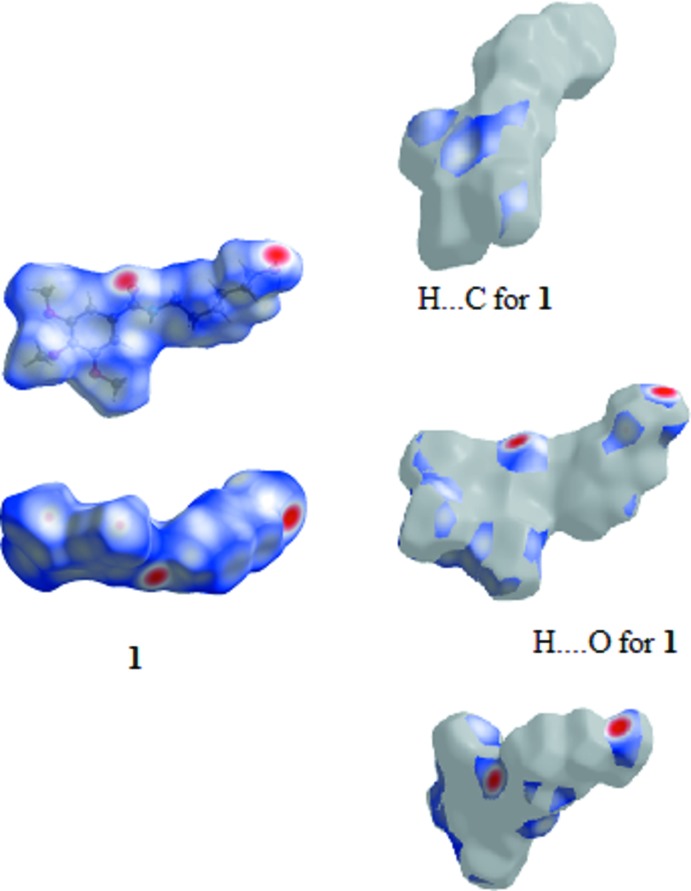
View of the Hirshfeld surface mapped over *d*_norm_ for **1**.

Hirshfeld surfaces were generated using *Crystal Explorer 3.1* (Wolff *et al.*, 2012 ▸) mapped over *d*_norm_ for the title compounds. The contact distances *d*_i_ and *d*_e_ from the Hirshfeld surface to the nearest atom inside and outside, respectively, were used to analyse the inter­molecular inter­actions through the mapping of *d*_norm_ and the plot of *d*_i_ versus *d*_e_ provides two-dimensional fingerprint plots (Rohl *et al.*, 2008 ▸) that are used to summarize those contacts. Figs. 7 ▸–9 ▸ ▸ are views of the Hirshfeld surfaces mapped over *d*_norm_ for **1**, **2** and **3** respectively. Since the mol­ecules have a six-atom alkyl chain, most of the contacts are H⋯H contacts. Leaving these aside, the remaining surface highlights the red areas that indicate contact points for the atoms participating in the (O/N/C)—H⋯O inter­molecular inter­actions. There are also significant contributions of C—H⋯C contacts, as can be visualized in the figures for each compound. The percentages of (O/N/C)—H⋯O and C—H⋯C contacts are listed in Table 7 ▸.

In all three compounds, red spots near the amide indicate the N(amide)—H⋯O hydrogen bonds that connect the amide groups in the classic fashion, making a *C*(4) chain for all compounds. In **2** and **3**, there are two pairs of red spots at the amide environment indicating that, in these structures, the carbonyl oxygen atom acts as the receptor for another H contact (the C6—H6⋯O11 contact).

The classical O(hy­droxy)–H⋯O hydrogen bond is located at the chain ‘tail’ in **1** and is identified by two red spots indicating that the oxygen atom O19 acts as donor and acceptor making the *C*(3) chain. The red spots in structure **2** show another two hydrogen bonds: one of these involves the amine nitro­gen atom of the end ‘tail’ phenyl­amine residue and the other also indicates the involvement of the *p*-meth­oxy group located at the tri­meth­oxy­benzamide ‘head’. This behaviour contrasts with that observed for **1** and **3**, in which the meth­oxy groups are not involved in classical hydrogen bonding.

**Figure 10:**
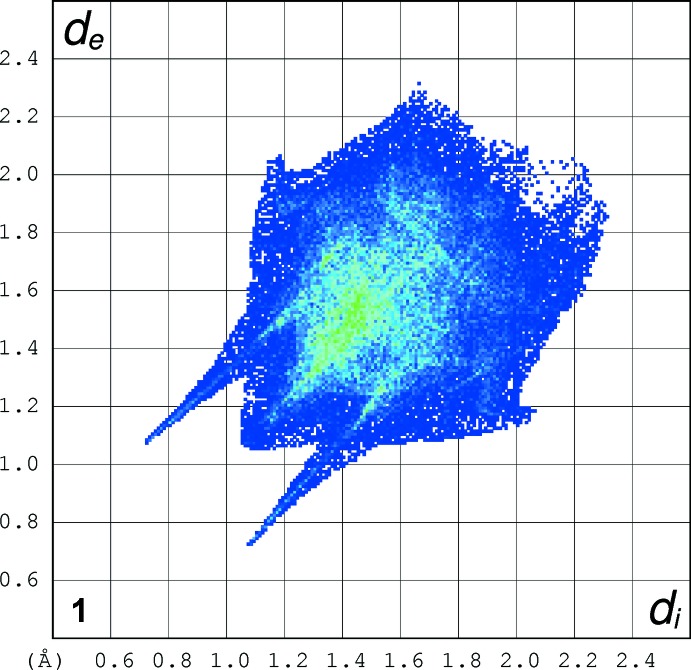
The full fingerprint (FP) plot showing various crystal packing inter­actions for **1**. Dark blue corresponds to the low frequency of occurrence of a *d*_i_/*d*_e_ pair, while light blue indicates a higher frequency for the occurrence.

The full fingerprint (FP) plots showing various crystal packing inter­actions are given in Figs. 10 ▸–12 ▸ ▸; the contributions from various contacts, listed in Table 7 ▸, were selected by the partial analysis of these plots. The FP plots show, for all compounds, a pair of long sharp spikes characteristic of a strong hydrogen bond, in an area near 1.7–1.8 Å. The symmetry of the upper left/down right spikes is an indication for the balance between the donor and acceptor character of the atoms involved in the hydrogen bonding, as seen before. They correspond to the N—H⋯O and O—H⋯O contacts. The *d*_e_/*d*_i_ points corresponding to H⋯H inter­actions appear around the hydrogen atom van der Waals radius of 1.20 Å. The wings in the graphical representation of **2** indicate that C—H⋯π inter­actions are more relevant in this crystal structure, highlighting the contribution of the C—H⋯π inter­action (Table 5 ▸) involving the phenyl­amide residue of the ‘tail’. Structure **2** also displays the biggest percentage of H⋯C/C⋯H contacts: besides the C—H⋯π contacts with the aromatic ring that define the supra­molecular structure for all compounds, in **2** the benzene ring of the phenyl­amine forms an extra inter­action of this kind

## Database survey   

A search made in the February 2016 version of the Cambridge Structural Database, (Groom *et al.*, 2016 ▸), revealed the existence of 37 structures (containing 48 unique mol­ecules) featuring the 3,4,5-trisubstituted benzamide scaffold.

*ortho-*C atom C2 was selected such that the amino N atom N12 was *trans* to it and in the following survey it is *trans-*related torsion angles which are discussed. The analysis of the torsion angles for the *o-*C atoms of the benzyl ring and the N atom of the benzamide group showed two distinct populations about 180° in the angular ranges −180 to −145° with a median value of −152.5° and 136–171° with a median value of 149.2°. The value of −179.3° for HESLEX, *N*,*N*-(heptane-2,6-di­yl)-*N′*-(3,4,5-meth­oxy­benzo­yl)thio­urea (Dillen *et al.*, 2006 ▸) is unusual: if this is excluded, then the lower limit for the negative range is −172°. The methyl groups attached to atoms C3 and C5 are inclined to the benzyl ring in the range −20 to 24° with a median values close to 0°. This excludes a mol­ecule with a C5 meth­oxy torsion angle of −85.9°: PIDTEC, 4-hy­droxy-3,5-di­eth­oxy­benzaldehyde-3,4,5-tri­meth­oxy­benzoylhydrazone monohydrate (Sun *et al.*, 2007 ▸). The methyl groups attached to atoms C4 are inclined to the benzyl ring in the ranges ±63 to ±122° with a median values close to ±90°. Of these 48 mol­ecules, 16 participate in N—H⋯O *C*(4) chains similar to those in the present compounds. In these structures, the torsion angles for the *trans o*-C atoms of the benzyl ring and the N atom of the benzamide group showed that, as above, the torsion angles lie in two populations: one in the range −153 to −145° and the other in the very similar positive range 142 to 165° with median values of −147.6° and 148.1°, respectively. The value for this torsion angle for **1**, −149.3 (3)° lies within the negative range, those for **2**, −167.27 (12)° and **3**, −158.58 (10)° lie outside this range.The results of the database searches are included in the supporting information.

## Synthesis and crystallization   

The title benzoic derivatives were obtained in moderate-to-high yields *via* the synthetic strategy described in the Scheme below. Compound **1** was obtained from 3,4,5-tri­meth­oxy­benz­oic acid by an amidation reaction using ethyl­chloro­formate as coupling agent. After oxidation of compound **1** alcohol function to an aldehyde, compounds (**2**) and (**3**) could be obtained. Compound **2** was synthesized by a reductive amination reaction using sodium tri­acet­oxy­boro­hydride as reducing agent. Compound **3** was synthesized using an ethano­lic solution of *N*-benzyl­hydroxyl­amine hydro­chlor­ide. 
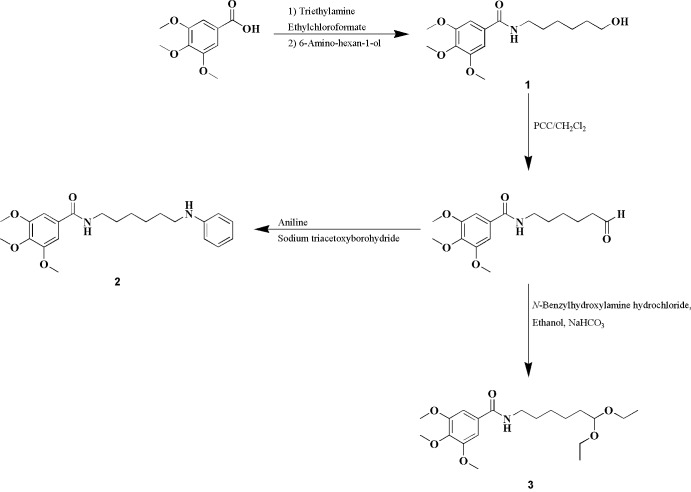


**1**: *N*-(6-hy­droxy­hex­yl)-3,4,5-tri­meth­oxy­benzamide **(1)**. Overall yield 82%; m.p. 393–399 K; crystallization solvent: ethyl acetate, to yield colourless needles.

**2**: *N*-(6-anilinohex­yl)-3,4,5-tri­meth­oxy­benzamide **(2)**. Overall yield 51%; m.p. 376–388 K; crystallization solvent: ethyl acetate to yield colourless laths

**3**: *N*-(6,6-di­eth­oxy­hex­yl)-3,4,5-tri­meth­oxy­benzamide **(3)**. Overall yield 50%; m.p. 364–374 K; crystallization solvents: chloro­form/*n*-hexane to yield colourless needles.

## Refinement   

Crystal data, data collection and structure refinement details are summarized in Table 8 ▸. The N—H and O—H hydrogen atoms were located in difference Fourier maps and freely refined. The C-bound H atoms were included in calculated positions and treated as riding: C—H(aromatic) = 0.95 Å and C—H2(methyl­ene) = 0.99 Å with *U*_iso_ = 1.2*U*_eq_(C), C—H(meth­yl) = 0.98 Å with *U*_iso_ = 1.5*U*_eq_(C).

## Supplementary Material

Crystal structure: contains datablock(s) 1, 2, 3, global. DOI: 10.1107/S2056989016005958/hb7575sup1.cif

Structure factors: contains datablock(s) 1. DOI: 10.1107/S2056989016005958/hb75751sup2.hkl

Structure factors: contains datablock(s) 2. DOI: 10.1107/S2056989016005958/hb75752sup3.hkl

Structure factors: contains datablock(s) 3. DOI: 10.1107/S2056989016005958/hb75753sup4.hkl

Supporting information file. DOI: 10.1107/S2056989016005958/hb75751sup5.cml

Supporting information file. DOI: 10.1107/S2056989016005958/hb75752sup6.cml

Supporting information file. DOI: 10.1107/S2056989016005958/hb75753sup7.cml

Supporting information file. DOI: 10.1107/S2056989016005958/hb7575sup8.pdf

Supporting information file. DOI: 10.1107/S2056989016005958/hb7575sup9.pdf

CCDC references: 1473261, 1473260, 1473259

Additional supporting information:  crystallographic information; 3D view; checkCIF report

## Figures and Tables

**Figure 2:**
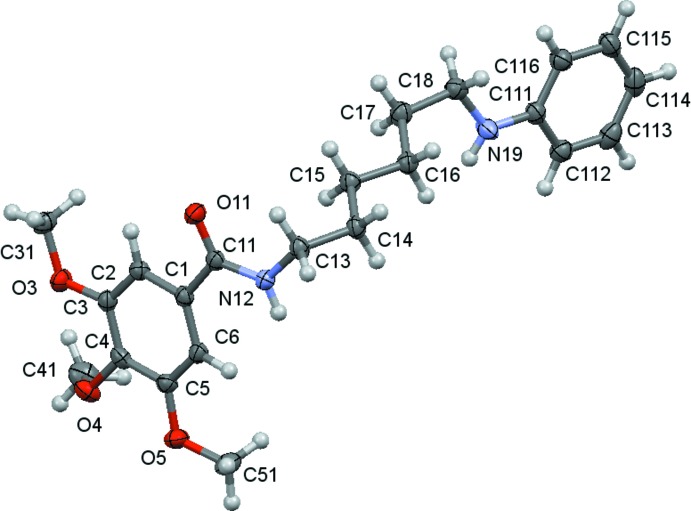
A view of the asymmetric unit of (**2**) with the atom-numbering scheme. Displacement ellipsoids are drawn at the 70% probability level.

**Figure 3:**
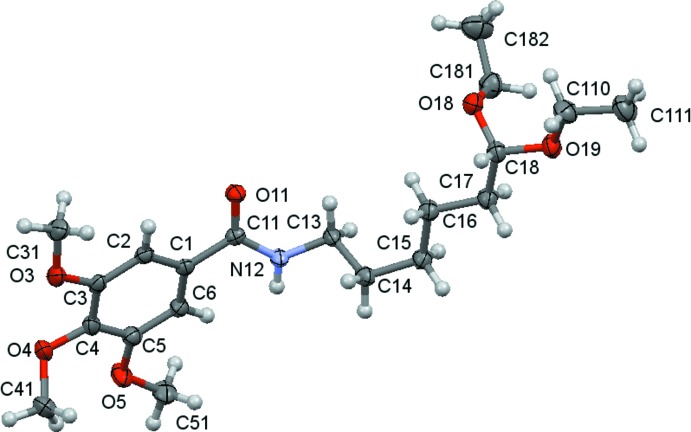
A view of the asymmetric unit of (**3**) with the atom-numbering scheme. Displacement ellipsoids are drawn at the 70% probability level.

**Figure 8:**
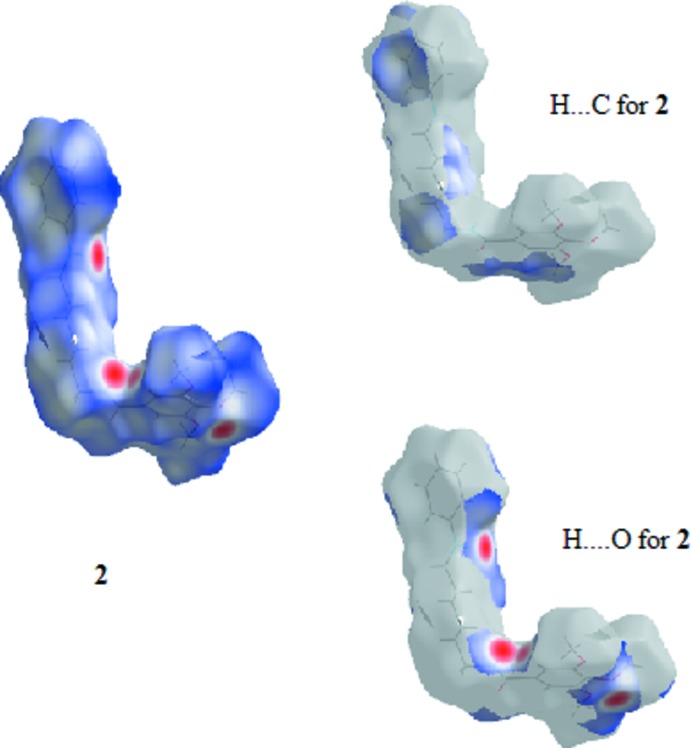
View of the Hirshfeld surface mapped over *d*_norm_ for **2**.

**Figure 9:**
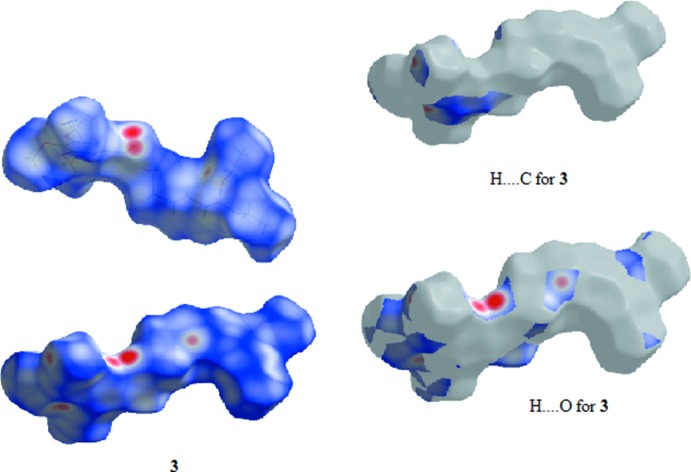
View of the Hirshfeld surface mapped over *d*_norm_ for **3**.

**Figure 11:**
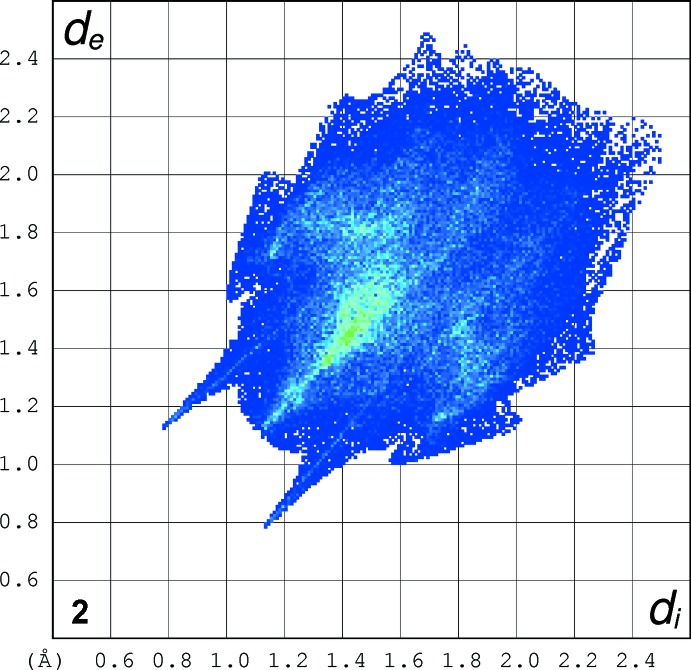
The full fingerprint (FP) plot showing various crystal packing inter­actions for **2**. Dark blue corresponds to the low frequency of occurrence of a *d*_i_/*d*_e_ pair, while light blue indicates a higher frequency for the occurrence.

**Figure 12:**
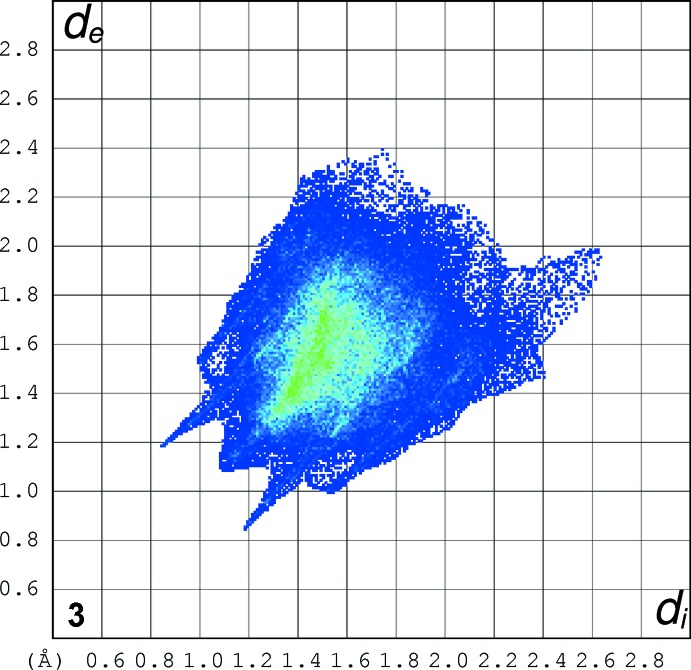
The full fingerprint (FP) plot showing various crystal packing inter­actions for **3**. Dark blue corresponds to the low frequency of occurrence of a *d*_i_/*d*_e_ pair, while light blue indicates a higher frequency for the occurrence.

**Table 1:** Selected torsion angles (°) for **1** [Chem scheme1]

C31—O3—C3—C4	176.7 (2)	C6—C1—C11—N12	35.6 (3)
C31—O3—C3—C2	−3.5 (4)	C11—N12—C13—C14	129.1 (3)
C41—O4—C4—C5	108.9 (3)	N12—C13—C14—C15	177.5 (2)
C41—O4—C4—C3	−74.4 (3)	C13—C14—C15—C16	65.7 (3)
C51—O5—C5—C4	−175.7 (2)	C14—C15—C16—C17	173.9 (2)
C51—O5—C5—C6	3.6 (4)	C15—C16—C17—C18	−174.4 (2)
C13—N12—C11—C1	−171.3 (2)	C16—C17—C18—O19	177.9 (2)
C2—C1—C11—N12	−149.3 (2)		

**Table 2:** Selected torsion angles (°) for **2** [Chem scheme1]

C31—O3—C3—C2	−0.16 (17)	C2—C1—C11—N12	−167.30 (11)
C31—O3—C3—C4	178.57 (11)	C11—N12—C13—C14	−112.80 (13)
C41—O4—C4—C3	67.59 (16)	N12—C13—C14—C15	66.85 (14)
C41—O4—C4—C5	−118.62 (13)	C13—C14—C15—C16	−179.75 (11)
C51—O5—C5—C6	−11.14 (18)	C14—C15—C16—C17	−175.06 (11)
C51—O5—C5—C4	170.38 (11)	C15—C16—C17—C18	175.02 (11)
C13—N12—C11—C1	179.22 (10)	C111—N19—C18—C17	172.76 (11)
C6—C1—C11—N12	13.05 (17)	C16—C17—C18—N19	67.90 (15)

**Table 3:** Selected torsion angles (°) for **3** [Chem scheme1]

C31—O3—C3—C2	9.59 (16)	C2—C1—C11—N12	158.58 (10)
C31—O3—C3—C4	−171.49 (10)	C6—C1—C11—N12	−19.07 (15)
C41—O4—C4—C5	61.51 (15)	C11—N12—C13—C14	114.65 (12)
C41—O4—C4—C3	−124.05 (12)	N12—C13—C14—C15	175.72 (9)
C51—O5—C5—C6	9.66 (17)	C13—C14—C15—C16	67.27 (13)
C51—O5—C5—C4	−171.35 (11)	C14—C15—C16—C17	175.71 (10)
C13—N12—C11—C1	−170.25 (10)	C15—C16—C17—C18	−177.76 (10)

**Table 4:** Hydrogen-bond geometry (Å, °) for **1** [Chem scheme1]

*D*—H⋯*A*	*D*—H	H⋯*A*	*D*⋯*A*	*D*—H⋯*A*
O19—H19⋯O19^i^	0.92 (4)	1.86 (4)	2.7799 (14)	176 (4)
N12—H12⋯O11^ii^	0.77 (3)	2.15 (3)	2.859 (3)	153 (3)
C18—H18*B*⋯O11^iii^	0.99	2.64	3.614 (3)	168
C41—H41*B*⋯O3	0.98	2.44	3.010 (3)	117

Symmetry codes: (i) 
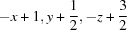
; (ii) 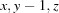; (iii) 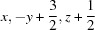.

**Table 5:** Hydrogen-bond geometry (Å, °) for **2** [Chem scheme1] *Cg* is the centroid of the C111–C116 ring.

*D*—H⋯*A*	*D*—H	H⋯*A*	*D*⋯*A*	*D*—H⋯*A*
N12—H12⋯O11^i^	0.867 (17)	2.052 (17)	2.9051 (14)	167.9 (15)
N19—H19⋯O4^i^	0.855 (17)	2.106 (17)	2.9436 (15)	166.3 (15)
C6—H6⋯O11^i^	0.95	2.33	3.2356 (15)	159
C41—H41*C*⋯O3	0.98	2.33	2.9287 (18)	119
C112—H112⋯O4^i^	0.95	2.65	3.3845 (16)	134
C13—H13*A*⋯*Cg* ^ii^	0.99	2.64	3.5272 (15)	148
C31—H31*C*⋯*Cg* ^iii^	0.98	2.62	3.5205 (16)	152

Symmetry codes: (i) 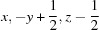; (ii) 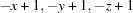; (iii) 
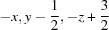
.

**Table 6:** Hydrogen-bond geometry (Å, °) for **3** [Chem scheme1]

*D*—H⋯*A*	*D*—H	H⋯*A*	*D*⋯*A*	*D*—H⋯*A*
N12—H12⋯O11^i^	0.856 (16)	2.169 (16)	2.9890 (13)	160.2 (14)
C6—H6⋯O11^i^	0.95	2.34	3.2549 (14)	162
C15—H15*B*⋯O18^ii^	0.99	2.49	3.4239 (14)	157

Symmetry codes: (i) 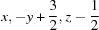; (ii) 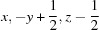.

**Table 7:** The percentages of (O/N/C)–H⋯O and C—H⋯C contacts

Contact	**1**	**2**	**3**
H⋯H	60.0	60.8	68.9
H⋯O/O⋯H	25.4	16.0	19.0
H⋯C/C⋯H	13.0	21.4	10.1
H⋯N/N⋯H	0.03	1.7	0.8

**Table 8:** Experimental details

	**1**	**2**	**3**
Crystal data			
Chemical formula	C_16_H_25_NO_5_	C_22_H_30_N_2_O_4_	C_20_H_33_NO_6_
*M*_r_	311.37	386.48	383.47
Crystal system, space group	Monoclinic, *P*2_1_/*c*	Monoclinic, *P*2_1_/*c*	Monoclinic, *P*2_1_/*c*
Temperature (K)	100	100	100
*a*, *b*, *c* (Å)	22.3351 (18), 5.0467 (4), 14.2265 (10)	11.5626 (8), 19.5328 (9), 9.5488 (7)	24.6345 (18), 8.4646 (5), 10.0598 (7)
β (°)	99.956 (7)	109.369 (8)	100.851 (2)
*V* (Å^3^)	1579.4 (2)	2034.5 (2)	2060.2 (2)
*Z*	4	4	4
Radiation type	Mo *K*α	Mo *K*α	Cu *K*α
μ (mm^−1^)	0.10	0.09	0.74
Crystal size (mm)	0.15 × 0.02 × 0.01	0.25 × 0.08 × 0.02	0.80 × 0.05 × 0.02

Data collection			
Diffractometer	Rigaku AFC12	Rigaku AFC12	Rigaku Saturn944+
Absorption correction	Multi-scan (*CrysAlis PRO*; Agilent, 2014 ▸)	Multi-scan (*CrysAlis PRO*; Agilent, 2014 ▸)	Multi-scan (*CrystalClear-SM Expert*; Rigaku, 2012 ▸)
*T*_min_, *T*_max_	0.803, 1.000	0.384, 1.000	0.814, 1.000
No. of measured, independent and observed [*I* > 2σ(*I*)] reflections	19396, 3627, 2039	26057, 4655, 3869	18993, 3706, 3362
*R*_int_	0.123	0.040	0.037
(sin θ/λ)_max_ (Å^−1^)	0.649	0.649	0.602

Refinement			
*R*[*F* ^2^ > 2σ(*F* ^2^)], *wR*(*F* ^2^), *S*	0.062, 0.133, 0.97	0.041, 0.105, 1.04	0.035, 0.095, 1.05
No. of reflections	3626	4652	3706
No. of parameters	210	264	253
H-atom treatment	H atoms treated by a mixture of independent and constrained refinement	H atoms treated by a mixture of independent and constrained refinement	H atoms treated by a mixture of independent and constrained refinement
Δρ_max_, Δρ_min_ (e Å^−3^)	0.25, −0.33	0.32, −0.18	0.23, −0.28

Computer programs: *CrystalClear-SM Expert* (Rigaku, 2012 ▸), *CrysAlis PRO* (Agilent, 2014 ▸), *SHELXT* (Sheldrick, 2015*a* ▸), *Flipper 25* (Oszlányi & Sütő, 2004 ▸), *OLEX2* (Dolomanov *et al.*, 2009 ▸), *OSCAIL* (McArdle *et al.*, 2004 ▸), *ShelXle* (Hübschle *et al.*, 2011 ▸), *SHELXL2014* (Sheldrick, 2015*b* ▸), *Mercury* (Macrae *et al.*, 2006 ▸) and *PLATON* (Spek, 2009 ▸).

## supplementary crystallographic information

### (1) N-(6-Hydroxyhexyl)-3,4,5-trimethoxybenzamide Crystal data

**Table:**

C_16_H_25_NO_5_	*F*(000) = 672
*M**_r_* = 311.37	*D*_x_ = 1.309 Mg m^−^^3^
Monoclinic, *P*2_1_/*c*	Mo *K*α radiation, λ = 0.71073 Å
*a* = 22.3351 (18) Å	Cell parameters from 4347 reflections
*b* = 5.0467 (4) Å	θ = 2.8–27.5°
*c* = 14.2265 (10) Å	µ = 0.10 mm^−^^1^
β = 99.956 (7)°	*T* = 100 K
*V* = 1579.4 (2) Å^3^	Needle, colourless
*Z* = 4	0.15 × 0.02 × 0.01 mm

### (1) N-(6-Hydroxyhexyl)-3,4,5-trimethoxybenzamide Data collection

**Table:**

Rigaku AFC12 diffractometer	3627 independent reflections
Radiation source: Rotating Anode	2039 reflections with *I* > 2σ(*I*)
Detector resolution: 28.5714 pixels mm^-1^	*R*_int_ = 0.123
profile data from ω–scans	θ_max_ = 27.5°, θ_min_ = 1.9°
Absorption correction: multi-scan (*CrysAlis PRO*; Agilent, 2014)	*h* = −29→28
*T*_min_ = 0.803, *T*_max_ = 1.000	*k* = −6→6
19396 measured reflections	*l* = −18→18

### (1) N-(6-Hydroxyhexyl)-3,4,5-trimethoxybenzamide Refinement

**Table:**

Refinement on *F*^2^	0 restraints
Least-squares matrix: full	Hydrogen site location: mixed
*R*[*F*^2^ > 2σ(*F*^2^)] = 0.062	H atoms treated by a mixture of independent and constrained refinement
*wR*(*F*^2^) = 0.133	*w* = 1/[σ^2^(*F*_o_^2^) + (0.0434*P*)^2^ + 1.2336*P*] where *P* = (*F*_o_^2^ + 2*F*_c_^2^)/3
*S* = 0.97	(Δ/σ)_max_ = 0.003
3626 reflections	Δρ_max_ = 0.25 e Å^−^^3^
210 parameters	Δρ_min_ = −0.33 e Å^−^^3^

### (1) N-(6-Hydroxyhexyl)-3,4,5-trimethoxybenzamide Special details

**Table:**

Geometry. All esds (except the esd in the dihedral angle between two l.s. planes) are estimated using the full covariance matrix. The cell esds are taken into account individually in the estimation of esds in distances, angles and torsion angles; correlations between esds in cell parameters are only used when they are defined by crystal symmetry. An approximate (isotropic) treatment of cell esds is used for estimating esds involving l.s. planes.

### (1) N-(6-Hydroxyhexyl)-3,4,5-trimethoxybenzamide Fractional atomic coordinates and isotropic or equivalent isotropic displacement parameters (Å^2^)

**Table:**

	*x*	*y*	*z*	*U*_iso_*/*U*_eq_	
O3	0.14707 (8)	0.7309 (4)	−0.17742 (12)	0.0226 (5)	
O4	0.06131 (7)	0.3824 (4)	−0.14864 (12)	0.0192 (4)	
O5	0.07387 (7)	0.0830 (4)	0.00766 (11)	0.0182 (4)	
O11	0.29716 (8)	0.7024 (4)	0.14247 (12)	0.0199 (4)	
O19	0.49154 (9)	0.5665 (4)	0.70885 (12)	0.0239 (5)	
H19	0.4980 (17)	0.729 (8)	0.738 (3)	0.071 (13)*	
N12	0.29857 (10)	0.2623 (5)	0.17360 (15)	0.0178 (5)	
H12	0.2877 (13)	0.124 (6)	0.154 (2)	0.026 (9)*	
C1	0.22016 (11)	0.4387 (5)	0.05130 (16)	0.0139 (6)	
C2	0.21185 (11)	0.6062 (5)	−0.02766 (16)	0.0156 (6)	
H2	0.2420	0.7331	−0.0355	0.019*	
C3	0.15876 (11)	0.5858 (5)	−0.09520 (16)	0.0160 (6)	
C4	0.11325 (11)	0.4077 (5)	−0.08137 (16)	0.0157 (6)	
C5	0.12136 (11)	0.2437 (5)	−0.00138 (17)	0.0144 (6)	
C6	0.17580 (11)	0.2542 (5)	0.06407 (17)	0.0152 (6)	
H6	0.1825	0.1363	0.1169	0.018*	
C11	0.27558 (11)	0.4775 (5)	0.12591 (17)	0.0143 (6)	
C13	0.34700 (11)	0.2710 (6)	0.25675 (17)	0.0186 (6)	
H13A	0.3551	0.4575	0.2766	0.022*	
H13B	0.3847	0.1966	0.2397	0.022*	
C14	0.32987 (11)	0.1155 (6)	0.33872 (17)	0.0188 (6)	
H14A	0.3197	−0.0682	0.3172	0.023*	
H14B	0.2930	0.1951	0.3568	0.023*	
C15	0.38004 (12)	0.1073 (5)	0.42678 (17)	0.0187 (6)	
H15A	0.3676	−0.0165	0.4739	0.022*	
H15B	0.4175	0.0357	0.4078	0.022*	
C16	0.39460 (12)	0.3745 (5)	0.47458 (18)	0.0183 (6)	
H16A	0.3566	0.4557	0.4879	0.022*	
H16B	0.4116	0.4932	0.4304	0.022*	
C17	0.43995 (11)	0.3505 (5)	0.56771 (17)	0.0178 (6)	
H17A	0.4248	0.2175	0.6090	0.021*	
H17B	0.4792	0.2856	0.5533	0.021*	
C18	0.45040 (12)	0.6094 (6)	0.62129 (17)	0.0200 (6)	
H18A	0.4676	0.7416	0.5820	0.024*	
H18B	0.4113	0.6787	0.6348	0.024*	
C31	0.19093 (12)	0.9255 (6)	−0.19180 (19)	0.0230 (7)	
H31A	0.1762	1.0234	−0.2508	0.034*	
H31B	0.2294	0.8380	−0.1968	0.034*	
H31C	0.1972	1.0487	−0.1377	0.034*	
C41	0.01986 (12)	0.6026 (6)	−0.14884 (19)	0.0222 (6)	
H41A	−0.0146	0.5805	−0.2011	0.033*	
H41B	0.0410	0.7684	−0.1577	0.033*	
H41C	0.0051	0.6081	−0.0879	0.033*	
C51	0.07898 (12)	−0.0741 (6)	0.09227 (18)	0.0216 (6)	
H51A	0.0404	−0.1652	0.0935	0.032*	
H51B	0.0887	0.0406	0.1484	0.032*	
H51C	0.1114	−0.2053	0.0929	0.032*	

### (1) N-(6-Hydroxyhexyl)-3,4,5-trimethoxybenzamide Atomic displacement parameters (Å^2^)

**Table:**

	*U*^11^	*U*^22^	*U*^33^	*U*^12^	*U*^13^	*U*^23^
O3	0.0264 (10)	0.0215 (12)	0.0182 (9)	−0.0036 (9)	−0.0009 (8)	0.0066 (8)
O4	0.0197 (10)	0.0133 (11)	0.0209 (9)	0.0013 (8)	−0.0071 (8)	−0.0020 (8)
O5	0.0166 (9)	0.0164 (11)	0.0200 (9)	−0.0060 (8)	−0.0013 (7)	0.0035 (8)
O11	0.0216 (10)	0.0107 (10)	0.0249 (10)	−0.0038 (8)	−0.0031 (8)	0.0000 (8)
O19	0.0322 (11)	0.0154 (12)	0.0186 (10)	0.0008 (9)	−0.0110 (8)	−0.0003 (9)
N12	0.0202 (12)	0.0120 (14)	0.0181 (12)	−0.0017 (11)	−0.0055 (9)	−0.0032 (10)
C1	0.0166 (13)	0.0096 (15)	0.0150 (12)	0.0015 (11)	0.0018 (10)	−0.0025 (10)
C2	0.0198 (14)	0.0115 (14)	0.0161 (12)	−0.0012 (12)	0.0052 (11)	0.0001 (11)
C3	0.0215 (14)	0.0137 (15)	0.0122 (12)	0.0035 (12)	0.0017 (10)	0.0014 (11)
C4	0.0175 (14)	0.0141 (14)	0.0136 (12)	0.0019 (12)	−0.0020 (10)	−0.0027 (11)
C5	0.0161 (13)	0.0105 (14)	0.0166 (13)	−0.0005 (11)	0.0028 (10)	−0.0014 (11)
C6	0.0192 (13)	0.0100 (14)	0.0164 (13)	0.0010 (11)	0.0031 (11)	0.0003 (11)
C11	0.0154 (13)	0.0102 (14)	0.0168 (13)	−0.0002 (11)	0.0015 (10)	−0.0022 (11)
C13	0.0187 (14)	0.0178 (16)	0.0166 (13)	−0.0016 (12)	−0.0045 (11)	0.0020 (11)
C14	0.0209 (14)	0.0163 (15)	0.0178 (13)	−0.0032 (12)	−0.0001 (11)	−0.0010 (12)
C15	0.0261 (15)	0.0130 (15)	0.0155 (13)	−0.0023 (12)	−0.0004 (11)	−0.0004 (11)
C16	0.0217 (14)	0.0140 (15)	0.0183 (13)	0.0001 (12)	0.0005 (11)	0.0004 (11)
C17	0.0205 (14)	0.0146 (16)	0.0175 (13)	−0.0004 (12)	0.0006 (11)	−0.0011 (11)
C18	0.0217 (14)	0.0203 (16)	0.0161 (13)	0.0000 (13)	−0.0018 (11)	−0.0006 (12)
C31	0.0238 (15)	0.0230 (17)	0.0229 (14)	0.0000 (13)	0.0064 (12)	0.0075 (13)
C41	0.0232 (15)	0.0157 (16)	0.0251 (14)	0.0015 (13)	−0.0031 (12)	0.0028 (12)
C51	0.0226 (14)	0.0184 (16)	0.0229 (14)	−0.0044 (13)	0.0017 (11)	0.0055 (12)

### (1) N-(6-Hydroxyhexyl)-3,4,5-trimethoxybenzamide Geometric parameters (Å, º)

**Table:**

O3—C3	1.366 (3)	C14—C15	1.530 (3)
O3—C31	1.427 (3)	C14—H14A	0.9900
O4—C4	1.376 (3)	C14—H14B	0.9900
O4—C41	1.446 (3)	C15—C16	1.520 (4)
O5—C5	1.359 (3)	C15—H15A	0.9900
O5—C51	1.429 (3)	C15—H15B	0.9900
O11—C11	1.240 (3)	C16—C17	1.527 (3)
O19—C18	1.431 (3)	C16—H16A	0.9900
O19—H19	0.92 (4)	C16—H16B	0.9900
N12—C11	1.335 (3)	C17—C18	1.510 (4)
N12—C13	1.459 (3)	C17—H17A	0.9900
N12—H12	0.77 (3)	C17—H17B	0.9900
C1—C2	1.393 (3)	C18—H18A	0.9900
C1—C6	1.394 (3)	C18—H18B	0.9900
C1—C11	1.498 (3)	C31—H31A	0.9800
C2—C3	1.395 (3)	C31—H31B	0.9800
C2—H2	0.9500	C31—H31C	0.9800
C3—C4	1.396 (4)	C41—H41A	0.9800
C4—C5	1.393 (3)	C41—H41B	0.9800
C5—C6	1.399 (3)	C41—H41C	0.9800
C6—H6	0.9500	C51—H51A	0.9800
C13—C14	1.509 (4)	C51—H51B	0.9800
C13—H13A	0.9900	C51—H51C	0.9800
C13—H13B	0.9900		
			
C3—O3—C31	117.22 (19)	C16—C15—H15A	108.7
C4—O4—C41	113.10 (19)	C14—C15—H15A	108.7
C5—O5—C51	117.44 (18)	C16—C15—H15B	108.7
C18—O19—H19	107 (2)	C14—C15—H15B	108.7
C11—N12—C13	123.6 (2)	H15A—C15—H15B	107.6
C11—N12—H12	119 (2)	C15—C16—C17	112.1 (2)
C13—N12—H12	117 (2)	C15—C16—H16A	109.2
C2—C1—C6	120.8 (2)	C17—C16—H16A	109.2
C2—C1—C11	118.1 (2)	C15—C16—H16B	109.2
C6—C1—C11	120.9 (2)	C17—C16—H16B	109.2
C1—C2—C3	119.3 (2)	H16A—C16—H16B	107.9
C1—C2—H2	120.3	C18—C17—C16	113.0 (2)
C3—C2—H2	120.3	C18—C17—H17A	109.0
O3—C3—C4	115.4 (2)	C16—C17—H17A	109.0
O3—C3—C2	124.3 (2)	C18—C17—H17B	109.0
C4—C3—C2	120.2 (2)	C16—C17—H17B	109.0
O4—C4—C5	119.2 (2)	H17A—C17—H17B	107.8
O4—C4—C3	120.6 (2)	O19—C18—C17	109.2 (2)
C5—C4—C3	120.2 (2)	O19—C18—H18A	109.8
O5—C5—C4	116.0 (2)	C17—C18—H18A	109.8
O5—C5—C6	124.2 (2)	O19—C18—H18B	109.8
C4—C5—C6	119.8 (2)	C17—C18—H18B	109.8
C1—C6—C5	119.6 (2)	H18A—C18—H18B	108.3
C1—C6—H6	120.2	O3—C31—H31A	109.5
C5—C6—H6	120.2	O3—C31—H31B	109.5
O11—C11—N12	123.1 (2)	H31A—C31—H31B	109.5
O11—C11—C1	120.1 (2)	O3—C31—H31C	109.5
N12—C11—C1	116.9 (2)	H31A—C31—H31C	109.5
N12—C13—C14	111.1 (2)	H31B—C31—H31C	109.5
N12—C13—H13A	109.4	O4—C41—H41A	109.5
C14—C13—H13A	109.4	O4—C41—H41B	109.5
N12—C13—H13B	109.4	H41A—C41—H41B	109.5
C14—C13—H13B	109.4	O4—C41—H41C	109.5
H13A—C13—H13B	108.0	H41A—C41—H41C	109.5
C13—C14—C15	113.5 (2)	H41B—C41—H41C	109.5
C13—C14—H14A	108.9	O5—C51—H51A	109.5
C15—C14—H14A	108.9	O5—C51—H51B	109.5
C13—C14—H14B	108.9	H51A—C51—H51B	109.5
C15—C14—H14B	108.9	O5—C51—H51C	109.5
H14A—C14—H14B	107.7	H51A—C51—H51C	109.5
C16—C15—C14	114.3 (2)	H51B—C51—H51C	109.5
			
C6—C1—C2—C3	−0.9 (4)	C3—C4—C5—C6	−1.3 (4)
C11—C1—C2—C3	−175.9 (2)	C2—C1—C6—C5	−2.3 (4)
C31—O3—C3—C4	176.7 (2)	C11—C1—C6—C5	172.6 (2)
C31—O3—C3—C2	−3.5 (4)	O5—C5—C6—C1	−175.8 (2)
C1—C2—C3—O3	−176.8 (2)	C4—C5—C6—C1	3.4 (4)
C1—C2—C3—C4	3.0 (4)	C13—N12—C11—O11	7.0 (4)
C41—O4—C4—C5	108.9 (3)	C13—N12—C11—C1	−171.3 (2)
C41—O4—C4—C3	−74.4 (3)	C2—C1—C11—O11	32.3 (4)
O3—C3—C4—O4	1.2 (4)	C6—C1—C11—O11	−142.8 (3)
C2—C3—C4—O4	−178.6 (2)	C2—C1—C11—N12	−149.3 (2)
O3—C3—C4—C5	177.9 (2)	C6—C1—C11—N12	35.6 (3)
C2—C3—C4—C5	−1.9 (4)	C11—N12—C13—C14	129.1 (3)
C51—O5—C5—C4	−175.7 (2)	N12—C13—C14—C15	177.5 (2)
C51—O5—C5—C6	3.6 (4)	C13—C14—C15—C16	65.7 (3)
O4—C4—C5—O5	−5.3 (4)	C14—C15—C16—C17	173.9 (2)
C3—C4—C5—O5	178.0 (2)	C15—C16—C17—C18	−174.4 (2)
O4—C4—C5—C6	175.5 (2)	C16—C17—C18—O19	177.9 (2)

### (1) N-(6-Hydroxyhexyl)-3,4,5-trimethoxybenzamide Hydrogen-bond geometry (Å, º)

**Table:**

*D*—H···*A*	*D*—H	H···*A*	*D*···*A*	*D*—H···*A*
O19—H19···O19^i^	0.92 (4)	1.86 (4)	2.7799 (14)	176 (4)
N12—H12···O11^ii^	0.77 (3)	2.15 (3)	2.859 (3)	153 (3)
C18—H18*B*···O11^iii^	0.99	2.64	3.614 (3)	168
C41—H41*B*···O3	0.98	2.44	3.010 (3)	117

Symmetry codes: (i) −*x*+1, *y*+1/2, −*z*+3/2; (ii) *x*, *y*−1, *z*; (iii) *x*, −*y*+3/2, *z*+1/2.

### (2) N-(6-Anilinohexyl)-3,4,5-trimethoxybenzamide Crystal data

**Table:**

C_22_H_30_N_2_O_4_	*F*(000) = 832
*M**_r_* = 386.48	*D*_x_ = 1.262 Mg m^−^^3^
Monoclinic, *P*2_1_/*c*	Mo *K*α radiation, λ = 0.71075 Å
*a* = 11.5626 (8) Å	Cell parameters from 12007 reflections
*b* = 19.5328 (9) Å	θ = 2.2–27.6°
*c* = 9.5488 (7) Å	µ = 0.09 mm^−^^1^
β = 109.369 (8)°	*T* = 100 K
*V* = 2034.5 (2) Å^3^	Lath, colourless
*Z* = 4	0.25 × 0.08 × 0.02 mm

### (2) N-(6-Anilinohexyl)-3,4,5-trimethoxybenzamide Data collection

**Table:**

Rigaku AFC12 diffractometer	4655 independent reflections
Radiation source: Rotating Anode	3869 reflections with *I* > 2σ(*I*)
Confocal mirrors, VHF Varimax monochromator	*R*_int_ = 0.040
Detector resolution: 28.5714 pixels mm^-1^	θ_max_ = 27.5°, θ_min_ = 1.9°
profile data from ω–scans	*h* = −15→15
Absorption correction: multi-scan (*CrysAlis PRO*; Agilent, 2014)	*k* = −25→25
*T*_min_ = 0.384, *T*_max_ = 1.000	*l* = −12→11
26057 measured reflections	

### (2) N-(6-Anilinohexyl)-3,4,5-trimethoxybenzamide Refinement

**Table:**

Refinement on *F*^2^	0 restraints
Least-squares matrix: full	Hydrogen site location: mixed
*R*[*F*^2^ > 2σ(*F*^2^)] = 0.041	H atoms treated by a mixture of independent and constrained refinement
*wR*(*F*^2^) = 0.105	*w* = 1/[σ^2^(*F*_o_^2^) + (0.0498*P*)^2^ + 0.7227*P*] where *P* = (*F*_o_^2^ + 2*F*_c_^2^)/3
*S* = 1.04	(Δ/σ)_max_ < 0.001
4652 reflections	Δρ_max_ = 0.32 e Å^−^^3^
264 parameters	Δρ_min_ = −0.17 e Å^−^^3^

### (2) N-(6-Anilinohexyl)-3,4,5-trimethoxybenzamide Special details

**Table:**

Geometry. All esds (except the esd in the dihedral angle between two l.s. planes) are estimated using the full covariance matrix. The cell esds are taken into account individually in the estimation of esds in distances, angles and torsion angles; correlations between esds in cell parameters are only used when they are defined by crystal symmetry. An approximate (isotropic) treatment of cell esds is used for estimating esds involving l.s. planes.

### (2) N-(6-Anilinohexyl)-3,4,5-trimethoxybenzamide Fractional atomic coordinates and isotropic or equivalent isotropic displacement parameters (Å^2^)

**Table:**

	*x*	*y*	*z*	*U*_iso_*/*U*_eq_	
O5	0.23589 (9)	0.06242 (5)	0.65347 (10)	0.0233 (2)	
O11	0.49022 (8)	0.29229 (5)	1.06168 (10)	0.0206 (2)	
O3	0.13699 (8)	0.14104 (5)	1.05838 (10)	0.0199 (2)	
O4	0.11195 (8)	0.05052 (4)	0.83767 (11)	0.0214 (2)	
N12	0.52046 (9)	0.27467 (5)	0.84328 (12)	0.0166 (2)	
H12	0.5016 (15)	0.2521 (8)	0.7605 (19)	0.025 (4)*	
N19	0.23085 (10)	0.58041 (6)	0.45509 (13)	0.0219 (2)	
H19	0.2032 (14)	0.5397 (9)	0.4356 (17)	0.024 (4)*	
C1	0.36942 (11)	0.20559 (6)	0.90565 (14)	0.0161 (2)	
C2	0.29676 (11)	0.20182 (6)	0.99581 (14)	0.0165 (2)	
H2	0.3076	0.2341	1.0735	0.020*	
C3	0.20868 (11)	0.15115 (6)	0.97263 (14)	0.0167 (3)	
C4	0.19028 (11)	0.10503 (6)	0.85609 (14)	0.0171 (3)	
C5	0.26120 (11)	0.10992 (6)	0.76358 (14)	0.0174 (3)	
C6	0.35187 (11)	0.15967 (6)	0.78929 (14)	0.0170 (3)	
H6	0.4015	0.1623	0.7277	0.020*	
C11	0.46561 (11)	0.26090 (6)	0.94280 (14)	0.0158 (2)	
C13	0.61401 (11)	0.32767 (6)	0.86549 (14)	0.0179 (3)	
H13A	0.6882	0.3072	0.8527	0.021*	
H13B	0.6367	0.3450	0.9685	0.021*	
C14	0.57161 (11)	0.38743 (6)	0.75829 (14)	0.0180 (3)	
H14A	0.6426	0.4178	0.7688	0.022*	
H14B	0.5435	0.3694	0.6556	0.022*	
C15	0.46909 (11)	0.42972 (6)	0.78092 (14)	0.0182 (3)	
H15A	0.4970	0.4483	0.8832	0.022*	
H15B	0.3978	0.3996	0.7704	0.022*	
C16	0.42906 (11)	0.48867 (6)	0.67131 (14)	0.0186 (3)	
H16A	0.5016	0.5166	0.6756	0.022*	
H16B	0.3942	0.4700	0.5695	0.022*	
C17	0.33424 (12)	0.53406 (7)	0.70390 (15)	0.0220 (3)	
H17A	0.2597	0.5065	0.6917	0.026*	
H17B	0.3669	0.5487	0.8090	0.026*	
C18	0.29791 (12)	0.59736 (6)	0.60736 (14)	0.0206 (3)	
H18A	0.2467	0.6272	0.6468	0.025*	
H18B	0.3726	0.6232	0.6116	0.025*	
C31	0.15479 (12)	0.18615 (7)	1.18125 (15)	0.0213 (3)	
H31A	0.1426	0.2335	1.1457	0.032*	
H31B	0.2383	0.1808	1.2510	0.032*	
H31C	0.0957	0.1751	1.2315	0.032*	
C41	−0.01587 (12)	0.06541 (8)	0.79790 (18)	0.0299 (3)	
H41A	−0.0621	0.0225	0.7856	0.045*	
H41B	−0.0427	0.0911	0.7045	0.045*	
H41C	−0.0305	0.0928	0.8764	0.045*	
C51	0.29185 (13)	0.07190 (7)	0.54292 (15)	0.0270 (3)	
H51A	0.2716	0.1175	0.4990	0.040*	
H51B	0.2615	0.0371	0.4655	0.040*	
H51C	0.3810	0.0676	0.5881	0.040*	
C111	0.17742 (11)	0.62986 (6)	0.35170 (14)	0.0182 (3)	
C112	0.10342 (11)	0.61135 (7)	0.20818 (14)	0.0207 (3)	
H112	0.0895	0.5643	0.1826	0.025*	
C113	0.05055 (12)	0.66108 (7)	0.10363 (15)	0.0237 (3)	
H113	0.0006	0.6478	0.0067	0.028*	
C114	0.06936 (12)	0.73016 (7)	0.13832 (16)	0.0248 (3)	
H114	0.0320	0.7642	0.0664	0.030*	
C115	0.14307 (12)	0.74837 (7)	0.27873 (16)	0.0241 (3)	
H115	0.1571	0.7955	0.3033	0.029*	
C116	0.19717 (12)	0.69942 (6)	0.38473 (15)	0.0206 (3)	
H116	0.2482	0.7132	0.4808	0.025*	

### (2) N-(6-Anilinohexyl)-3,4,5-trimethoxybenzamide Atomic displacement parameters (Å^2^)

**Table:**

	*U*^11^	*U*^22^	*U*^33^	*U*^12^	*U*^13^	*U*^23^
O5	0.0305 (5)	0.0196 (5)	0.0232 (5)	−0.0041 (4)	0.0135 (4)	−0.0067 (4)
O11	0.0256 (5)	0.0205 (5)	0.0192 (5)	−0.0044 (4)	0.0119 (4)	−0.0032 (4)
O3	0.0215 (4)	0.0213 (5)	0.0210 (5)	−0.0044 (4)	0.0123 (4)	−0.0027 (4)
O4	0.0191 (4)	0.0145 (4)	0.0320 (5)	−0.0016 (3)	0.0101 (4)	−0.0009 (4)
N12	0.0199 (5)	0.0155 (5)	0.0164 (6)	−0.0004 (4)	0.0087 (4)	0.0000 (4)
N19	0.0273 (6)	0.0138 (5)	0.0216 (6)	−0.0013 (4)	0.0039 (5)	0.0010 (4)
C1	0.0176 (5)	0.0150 (6)	0.0162 (6)	0.0027 (4)	0.0062 (5)	0.0036 (5)
C2	0.0188 (6)	0.0154 (6)	0.0159 (6)	0.0014 (5)	0.0066 (5)	0.0002 (5)
C3	0.0176 (6)	0.0168 (6)	0.0175 (6)	0.0030 (5)	0.0083 (5)	0.0036 (5)
C4	0.0171 (6)	0.0125 (6)	0.0213 (7)	0.0004 (4)	0.0059 (5)	0.0028 (5)
C5	0.0210 (6)	0.0146 (6)	0.0165 (6)	0.0035 (5)	0.0060 (5)	−0.0001 (5)
C6	0.0198 (6)	0.0169 (6)	0.0165 (6)	0.0025 (5)	0.0091 (5)	0.0022 (5)
C11	0.0175 (5)	0.0148 (6)	0.0161 (6)	0.0033 (4)	0.0068 (5)	0.0025 (5)
C13	0.0170 (6)	0.0175 (6)	0.0212 (7)	0.0002 (5)	0.0090 (5)	0.0029 (5)
C14	0.0194 (6)	0.0165 (6)	0.0203 (7)	−0.0004 (5)	0.0095 (5)	0.0029 (5)
C15	0.0186 (6)	0.0174 (6)	0.0202 (7)	0.0003 (5)	0.0085 (5)	0.0019 (5)
C16	0.0202 (6)	0.0180 (6)	0.0184 (7)	0.0012 (5)	0.0076 (5)	0.0017 (5)
C17	0.0237 (6)	0.0231 (6)	0.0212 (7)	0.0053 (5)	0.0098 (5)	0.0044 (5)
C18	0.0229 (6)	0.0183 (6)	0.0207 (7)	0.0029 (5)	0.0075 (5)	0.0000 (5)
C31	0.0209 (6)	0.0254 (7)	0.0213 (7)	−0.0025 (5)	0.0120 (5)	−0.0036 (5)
C41	0.0184 (6)	0.0284 (7)	0.0415 (9)	−0.0027 (5)	0.0082 (6)	−0.0028 (6)
C51	0.0351 (7)	0.0280 (7)	0.0206 (7)	−0.0021 (6)	0.0130 (6)	−0.0066 (6)
C111	0.0177 (6)	0.0175 (6)	0.0217 (7)	0.0013 (5)	0.0097 (5)	0.0019 (5)
C112	0.0218 (6)	0.0207 (6)	0.0217 (7)	0.0000 (5)	0.0100 (5)	−0.0014 (5)
C113	0.0205 (6)	0.0330 (7)	0.0195 (7)	0.0026 (5)	0.0090 (5)	0.0014 (6)
C114	0.0241 (6)	0.0274 (7)	0.0270 (8)	0.0075 (5)	0.0139 (6)	0.0109 (6)
C115	0.0277 (7)	0.0175 (6)	0.0316 (8)	0.0030 (5)	0.0157 (6)	0.0049 (5)
C116	0.0224 (6)	0.0179 (6)	0.0229 (7)	0.0004 (5)	0.0093 (5)	−0.0001 (5)

### (2) N-(6-Anilinohexyl)-3,4,5-trimethoxybenzamide Geometric parameters (Å, º)

**Table:**

O5—C5	1.3594 (15)	C15—H15B	0.9900
O5—C51	1.4214 (16)	C16—C17	1.5204 (17)
O11—C11	1.2374 (15)	C16—H16A	0.9900
O3—C3	1.3590 (14)	C16—H16B	0.9900
O3—C31	1.4266 (15)	C17—C18	1.5159 (18)
O4—C4	1.3709 (14)	C17—H17A	0.9900
O4—C41	1.4286 (15)	C17—H17B	0.9900
N12—C11	1.3332 (15)	C18—H18A	0.9900
N12—C13	1.4610 (15)	C18—H18B	0.9900
N12—H12	0.867 (17)	C31—H31A	0.9800
N19—C111	1.3729 (17)	C31—H31B	0.9800
N19—C18	1.4412 (17)	C31—H31C	0.9800
N19—H19	0.855 (17)	C41—H41A	0.9800
C1—C6	1.3894 (17)	C41—H41B	0.9800
C1—C2	1.3901 (16)	C41—H41C	0.9800
C1—C11	1.5061 (17)	C51—H51A	0.9800
C2—C3	1.3842 (17)	C51—H51B	0.9800
C2—H2	0.9500	C51—H51C	0.9800
C3—C4	1.3922 (17)	C111—C116	1.3963 (17)
C4—C5	1.3937 (17)	C111—C112	1.4011 (18)
C5—C6	1.3903 (17)	C112—C113	1.3816 (19)
C6—H6	0.9500	C112—H112	0.9500
C13—C14	1.5224 (17)	C113—C114	1.389 (2)
C13—H13A	0.9900	C113—H113	0.9500
C13—H13B	0.9900	C114—C115	1.376 (2)
C14—C15	1.5179 (16)	C114—H114	0.9500
C14—H14A	0.9900	C115—C116	1.3821 (19)
C14—H14B	0.9900	C115—H115	0.9500
C15—C16	1.5212 (17)	C116—H116	0.9500
C15—H15A	0.9900		
			
C5—O5—C51	116.83 (10)	C17—C16—H16B	109.2
C3—O3—C31	117.08 (10)	C15—C16—H16B	109.2
C4—O4—C41	117.21 (10)	H16A—C16—H16B	107.9
C11—N12—C13	122.94 (11)	C18—C17—C16	115.11 (11)
C11—N12—H12	120.8 (11)	C18—C17—H17A	108.5
C13—N12—H12	116.3 (11)	C16—C17—H17A	108.5
C111—N19—C18	121.81 (11)	C18—C17—H17B	108.5
C111—N19—H19	116.8 (10)	C16—C17—H17B	108.5
C18—N19—H19	118.1 (10)	H17A—C17—H17B	107.5
C6—C1—C2	120.20 (11)	N19—C18—C17	111.97 (11)
C6—C1—C11	123.51 (11)	N19—C18—H18A	109.2
C2—C1—C11	116.29 (11)	C17—C18—H18A	109.2
C3—C2—C1	120.18 (11)	N19—C18—H18B	109.2
C3—C2—H2	119.9	C17—C18—H18B	109.2
C1—C2—H2	119.9	H18A—C18—H18B	107.9
O3—C3—C2	124.83 (11)	O3—C31—H31A	109.5
O3—C3—C4	115.18 (11)	O3—C31—H31B	109.5
C2—C3—C4	119.98 (11)	H31A—C31—H31B	109.5
O4—C4—C3	121.63 (11)	O3—C31—H31C	109.5
O4—C4—C5	118.32 (11)	H31A—C31—H31C	109.5
C3—C4—C5	119.75 (11)	H31B—C31—H31C	109.5
O5—C5—C6	124.75 (11)	O4—C41—H41A	109.5
O5—C5—C4	114.99 (11)	O4—C41—H41B	109.5
C6—C5—C4	120.24 (11)	H41A—C41—H41B	109.5
C1—C6—C5	119.60 (11)	O4—C41—H41C	109.5
C1—C6—H6	120.2	H41A—C41—H41C	109.5
C5—C6—H6	120.2	H41B—C41—H41C	109.5
O11—C11—N12	122.34 (11)	O5—C51—H51A	109.5
O11—C11—C1	119.91 (11)	O5—C51—H51B	109.5
N12—C11—C1	117.74 (11)	H51A—C51—H51B	109.5
N12—C13—C14	112.83 (10)	O5—C51—H51C	109.5
N12—C13—H13A	109.0	H51A—C51—H51C	109.5
C14—C13—H13A	109.0	H51B—C51—H51C	109.5
N12—C13—H13B	109.0	N19—C111—C116	121.42 (12)
C14—C13—H13B	109.0	N19—C111—C112	120.33 (12)
H13A—C13—H13B	107.8	C116—C111—C112	118.23 (12)
C15—C14—C13	114.46 (10)	C113—C112—C111	120.38 (12)
C15—C14—H14A	108.6	C113—C112—H112	119.8
C13—C14—H14A	108.6	C111—C112—H112	119.8
C15—C14—H14B	108.6	C112—C113—C114	120.91 (13)
C13—C14—H14B	108.6	C112—C113—H113	119.5
H14A—C14—H14B	107.6	C114—C113—H113	119.5
C14—C15—C16	112.81 (10)	C115—C114—C113	118.74 (12)
C14—C15—H15A	109.0	C115—C114—H114	120.6
C16—C15—H15A	109.0	C113—C114—H114	120.6
C14—C15—H15B	109.0	C114—C115—C116	121.23 (13)
C16—C15—H15B	109.0	C114—C115—H115	119.4
H15A—C15—H15B	107.8	C116—C115—H115	119.4
C17—C16—C15	112.09 (10)	C115—C116—C111	120.49 (13)
C17—C16—H16A	109.2	C115—C116—H116	119.8
C15—C16—H16A	109.2	C111—C116—H116	119.8
			
C6—C1—C2—C3	1.73 (18)	C13—N12—C11—C1	179.22 (10)
C11—C1—C2—C3	−177.93 (11)	C6—C1—C11—O11	−167.75 (11)
C31—O3—C3—C2	−0.16 (17)	C2—C1—C11—O11	11.89 (17)
C31—O3—C3—C4	178.57 (11)	C6—C1—C11—N12	13.05 (17)
C1—C2—C3—O3	176.80 (11)	C2—C1—C11—N12	−167.30 (11)
C1—C2—C3—C4	−1.87 (18)	C11—N12—C13—C14	−112.80 (13)
C41—O4—C4—C3	67.59 (16)	N12—C13—C14—C15	66.85 (14)
C41—O4—C4—C5	−118.62 (13)	C13—C14—C15—C16	−179.75 (11)
O3—C3—C4—O4	−4.78 (17)	C14—C15—C16—C17	−175.06 (11)
C2—C3—C4—O4	174.01 (11)	C15—C16—C17—C18	175.02 (11)
O3—C3—C4—C5	−178.49 (11)	C111—N19—C18—C17	172.76 (11)
C2—C3—C4—C5	0.30 (18)	C16—C17—C18—N19	67.90 (15)
C51—O5—C5—C6	−11.14 (18)	C18—N19—C111—C116	7.44 (18)
C51—O5—C5—C4	170.38 (11)	C18—N19—C111—C112	−174.08 (11)
O4—C4—C5—O5	6.04 (16)	N19—C111—C112—C113	−179.39 (12)
C3—C4—C5—O5	179.96 (11)	C116—C111—C112—C113	−0.86 (18)
O4—C4—C5—C6	−172.51 (11)	C111—C112—C113—C114	−0.06 (19)
C3—C4—C5—C6	1.41 (18)	C112—C113—C114—C115	0.76 (19)
C2—C1—C6—C5	−0.02 (18)	C113—C114—C115—C116	−0.52 (19)
C11—C1—C6—C5	179.62 (11)	C114—C115—C116—C111	−0.41 (19)
O5—C5—C6—C1	−179.95 (11)	N19—C111—C116—C115	179.61 (12)
C4—C5—C6—C1	−1.55 (18)	C112—C111—C116—C115	1.09 (18)
C13—N12—C11—O11	0.05 (18)		

### (2) N-(6-Anilinohexyl)-3,4,5-trimethoxybenzamide Hydrogen-bond geometry (Å, º)

Cg is the centroid of the C111–C116 ring.

**Table:**

*D*—H···*A*	*D*—H	H···*A*	*D*···*A*	*D*—H···*A*
N12—H12···O11^i^	0.867 (17)	2.052 (17)	2.9051 (14)	167.9 (15)
N19—H19···O4^i^	0.855 (17)	2.106 (17)	2.9436 (15)	166.3 (15)
C6—H6···O11^i^	0.95	2.33	3.2356 (15)	159
C41—H41*C*···O3	0.98	2.33	2.9287 (18)	119
C112—H112···O4^i^	0.95	2.65	3.3845 (16)	134
C13—H13*A*···*Cg*^ii^	0.99	2.64	3.5272 (15)	148
C31—H31*C*···*Cg*^iii^	0.98	2.62	3.5205 (16)	152

Symmetry codes: (i) *x*, −*y*+1/2, *z*−1/2; (ii) −*x*+1, −*y*+1, −*z*+1; (iii) −*x*, *y*−1/2, −*z*+3/2.

### (3) N-(6,6-Diethoxyhexyl)-3,4,5-trimethoxybenzamide Crystal data

**Table:**

C_20_H_33_NO_6_	*F*(000) = 832
*M**_r_* = 383.47	*D*_x_ = 1.236 Mg m^−^^3^
Monoclinic, *P*2_1_/*c*	Cu *K*α radiation, λ = 1.5418 Å
*a* = 24.6345 (18) Å	Cell parameters from 18993 reflections
*b* = 8.4646 (5) Å	θ = 3.7–68.3°
*c* = 10.0598 (7) Å	µ = 0.74 mm^−^^1^
β = 100.851 (2)°	*T* = 100 K
*V* = 2060.2 (2) Å^3^	Needle, colourless
*Z* = 4	0.80 × 0.05 × 0.02 mm

### (3) N-(6,6-Diethoxyhexyl)-3,4,5-trimethoxybenzamide Data collection

**Table:**

Rigaku Saturn944+ diffractometer	3706 independent reflections
Radiation source: Sealed Tube	3362 reflections with *I* > 2σ(*I*)
Confocal monochromator	*R*_int_ = 0.037
Detector resolution: 22.2222 pixels mm^-1^	θ_max_ = 68.2°, θ_min_ = 3.7°
profile data from ω–scans	*h* = −29→28
Absorption correction: multi-scan (*CrystalClear-SM Expert*; Rigaku, 2012)	*k* = −10→10
*T*_min_ = 0.814, *T*_max_ = 1.000	*l* = −8→11
18993 measured reflections	

### (3) N-(6,6-Diethoxyhexyl)-3,4,5-trimethoxybenzamide Refinement

**Table:**

Refinement on *F*^2^	0 restraints
Least-squares matrix: full	Hydrogen site location: mixed
*R*[*F*^2^ > 2σ(*F*^2^)] = 0.035	H atoms treated by a mixture of independent and constrained refinement
*wR*(*F*^2^) = 0.095	*w* = 1/[σ^2^(*F*_o_^2^) + (0.0504*P*)^2^ + 0.704*P*] where *P* = (*F*_o_^2^ + 2*F*_c_^2^)/3
*S* = 1.05	(Δ/σ)_max_ = 0.004
3706 reflections	Δρ_max_ = 0.23 e Å^−^^3^
253 parameters	Δρ_min_ = −0.28 e Å^−^^3^

### (3) N-(6,6-Diethoxyhexyl)-3,4,5-trimethoxybenzamide Special details

**Table:**

Geometry. All esds (except the esd in the dihedral angle between two l.s. planes) are estimated using the full covariance matrix. The cell esds are taken into account individually in the estimation of esds in distances, angles and torsion angles; correlations between esds in cell parameters are only used when they are defined by crystal symmetry. An approximate (isotropic) treatment of cell esds is used for estimating esds involving l.s. planes.

### (3) N-(6,6-Diethoxyhexyl)-3,4,5-trimethoxybenzamide Fractional atomic coordinates and isotropic or equivalent isotropic displacement parameters (Å^2^)

**Table:**

	*x*	*y*	*z*	*U*_iso_*/*U*_eq_	
O3	0.06047 (3)	1.01933 (10)	0.47561 (8)	0.0216 (2)	
O4	0.04431 (3)	1.17888 (10)	0.24751 (9)	0.0238 (2)	
O5	0.11083 (4)	1.13704 (10)	0.06165 (9)	0.0262 (2)	
O11	0.23438 (3)	0.69400 (9)	0.48281 (8)	0.01775 (19)	
O18	0.38282 (3)	0.03224 (10)	0.56738 (8)	0.0217 (2)	
O19	0.38224 (3)	−0.19607 (9)	0.43634 (8)	0.0226 (2)	
N12	0.23993 (4)	0.67500 (11)	0.26098 (10)	0.0171 (2)	
H12	0.2298 (6)	0.7133 (18)	0.1814 (16)	0.026 (4)*	
C1	0.17484 (4)	0.85971 (13)	0.32763 (11)	0.0151 (2)	
C2	0.14072 (4)	0.88484 (13)	0.42145 (11)	0.0161 (2)	
H2	0.1476	0.8305	0.5056	0.019*	
C3	0.09662 (5)	0.98949 (13)	0.39175 (12)	0.0171 (2)	
C4	0.08646 (5)	1.07014 (13)	0.26822 (12)	0.0189 (3)	
C5	0.12200 (5)	1.04740 (13)	0.17665 (12)	0.0192 (3)	
C6	0.16583 (5)	0.94151 (13)	0.20520 (12)	0.0171 (2)	
H6	0.1894	0.9251	0.1418	0.020*	
C11	0.21940 (4)	0.73742 (13)	0.36342 (11)	0.0144 (2)	
C13	0.27693 (5)	0.53875 (13)	0.27617 (12)	0.0179 (2)	
H13A	0.2870	0.5098	0.3730	0.021*	
H13B	0.3113	0.5663	0.2437	0.021*	
C14	0.24913 (5)	0.39825 (14)	0.19562 (12)	0.0191 (3)	
H14A	0.2363	0.4312	0.1005	0.023*	
H14B	0.2162	0.3672	0.2328	0.023*	
C15	0.28700 (5)	0.25481 (13)	0.19823 (12)	0.0191 (3)	
H15A	0.2683	0.1758	0.1327	0.023*	
H15B	0.3211	0.2882	0.1675	0.023*	
C16	0.30308 (5)	0.17604 (13)	0.33596 (12)	0.0200 (3)	
H16A	0.2693	0.1481	0.3705	0.024*	
H16B	0.3248	0.2510	0.4005	0.024*	
C17	0.33722 (5)	0.02709 (14)	0.32709 (12)	0.0201 (3)	
H17A	0.3159	−0.0450	0.2591	0.024*	
H17B	0.3715	0.0566	0.2952	0.024*	
C18	0.35258 (5)	−0.06007 (13)	0.46037 (12)	0.0195 (3)	
H18	0.3176	−0.0952	0.4882	0.023*	
C31	0.07377 (5)	0.95685 (17)	0.60957 (12)	0.0262 (3)	
H31A	0.0722	0.8412	0.6058	0.039*	
H31B	0.0472	0.9961	0.6629	0.039*	
H31C	0.1111	0.9903	0.6520	0.039*	
C41	0.00213 (5)	1.15608 (16)	0.12914 (13)	0.0277 (3)	
H41A	0.0158	1.1922	0.0489	0.042*	
H41B	−0.0308	1.2168	0.1384	0.042*	
H41C	−0.0073	1.0437	0.1195	0.042*	
C51	0.14099 (6)	1.10167 (16)	−0.04275 (13)	0.0270 (3)	
H51A	0.1373	0.9890	−0.0651	0.041*	
H51B	0.1801	1.1273	−0.0114	0.041*	
H51C	0.1263	1.1644	−0.1234	0.041*	
C110	0.38838 (6)	−0.30686 (15)	0.54572 (13)	0.0267 (3)	
H11A	0.3517	−0.3427	0.5601	0.032*	
H11B	0.4081	−0.2572	0.6303	0.032*	
C111	0.42100 (6)	−0.44433 (16)	0.50818 (15)	0.0331 (3)	
H11C	0.4018	−0.4900	0.4227	0.050*	
H11D	0.4248	−0.5245	0.5796	0.050*	
H11E	0.4578	−0.4081	0.4978	0.050*	
C181	0.43434 (5)	0.09299 (16)	0.54379 (13)	0.0272 (3)	
H18A	0.4279	0.1870	0.4843	0.033*	
H18B	0.4536	0.0120	0.4988	0.033*	
C182	0.46903 (6)	0.13715 (18)	0.67851 (15)	0.0368 (3)	
H18C	0.5043	0.1814	0.6645	0.055*	
H18D	0.4760	0.0429	0.7357	0.055*	
H18E	0.4493	0.2159	0.7229	0.055*	

### (3) N-(6,6-Diethoxyhexyl)-3,4,5-trimethoxybenzamide Atomic displacement parameters (Å^2^)

**Table:**

	*U*^11^	*U*^22^	*U*^33^	*U*^12^	*U*^13^	*U*^23^
O3	0.0169 (4)	0.0288 (5)	0.0197 (4)	0.0044 (3)	0.0051 (3)	−0.0042 (3)
O4	0.0213 (4)	0.0219 (4)	0.0258 (5)	0.0092 (3)	−0.0019 (4)	−0.0044 (3)
O5	0.0329 (5)	0.0246 (4)	0.0216 (5)	0.0120 (4)	0.0065 (4)	0.0075 (3)
O11	0.0182 (4)	0.0221 (4)	0.0131 (4)	0.0027 (3)	0.0032 (3)	0.0022 (3)
O18	0.0215 (4)	0.0241 (4)	0.0199 (4)	0.0029 (3)	0.0046 (3)	−0.0016 (3)
O19	0.0260 (5)	0.0182 (4)	0.0241 (5)	0.0061 (3)	0.0057 (4)	0.0015 (3)
N12	0.0197 (5)	0.0192 (5)	0.0127 (5)	0.0055 (4)	0.0035 (4)	0.0021 (4)
C1	0.0139 (5)	0.0148 (5)	0.0158 (6)	−0.0014 (4)	0.0005 (4)	−0.0028 (4)
C2	0.0157 (5)	0.0174 (5)	0.0146 (6)	−0.0016 (4)	0.0014 (4)	−0.0022 (4)
C3	0.0146 (5)	0.0181 (5)	0.0185 (6)	−0.0017 (4)	0.0029 (4)	−0.0067 (4)
C4	0.0173 (6)	0.0153 (5)	0.0228 (6)	0.0027 (4)	0.0002 (5)	−0.0039 (4)
C5	0.0229 (6)	0.0160 (5)	0.0174 (6)	0.0012 (4)	0.0003 (5)	0.0003 (4)
C6	0.0184 (6)	0.0170 (5)	0.0159 (6)	0.0004 (4)	0.0035 (4)	−0.0012 (4)
C11	0.0141 (5)	0.0148 (5)	0.0144 (6)	−0.0029 (4)	0.0031 (4)	−0.0001 (4)
C13	0.0185 (6)	0.0195 (6)	0.0159 (6)	0.0056 (4)	0.0035 (4)	0.0014 (4)
C14	0.0193 (6)	0.0206 (6)	0.0170 (6)	0.0028 (5)	0.0027 (4)	0.0020 (5)
C15	0.0220 (6)	0.0181 (6)	0.0176 (6)	0.0016 (5)	0.0047 (5)	−0.0002 (4)
C16	0.0230 (6)	0.0192 (6)	0.0184 (6)	0.0023 (5)	0.0052 (5)	0.0012 (5)
C17	0.0220 (6)	0.0195 (6)	0.0197 (6)	0.0021 (5)	0.0057 (5)	−0.0003 (5)
C18	0.0192 (6)	0.0183 (6)	0.0215 (6)	0.0031 (4)	0.0047 (5)	0.0000 (5)
C31	0.0202 (6)	0.0405 (7)	0.0187 (6)	0.0043 (5)	0.0056 (5)	−0.0041 (5)
C41	0.0209 (6)	0.0321 (7)	0.0272 (7)	0.0078 (5)	−0.0029 (5)	−0.0017 (5)
C51	0.0333 (7)	0.0288 (6)	0.0192 (6)	0.0083 (5)	0.0057 (5)	0.0070 (5)
C110	0.0304 (7)	0.0231 (6)	0.0253 (7)	0.0039 (5)	0.0021 (5)	0.0056 (5)
C111	0.0338 (8)	0.0244 (7)	0.0391 (8)	0.0077 (6)	0.0014 (6)	0.0037 (6)
C181	0.0264 (7)	0.0269 (6)	0.0292 (7)	−0.0011 (5)	0.0078 (5)	−0.0020 (5)
C182	0.0327 (8)	0.0382 (8)	0.0360 (8)	−0.0058 (6)	−0.0028 (6)	0.0007 (6)

### (3) N-(6,6-Diethoxyhexyl)-3,4,5-trimethoxybenzamide Geometric parameters (Å, º)

**Table:**

O3—C3	1.3606 (14)	C15—H15A	0.9900
O3—C31	1.4272 (15)	C15—H15B	0.9900
O4—C4	1.3738 (14)	C16—C17	1.5274 (15)
O4—C41	1.4385 (15)	C16—H16A	0.9900
O5—C5	1.3675 (14)	C16—H16B	0.9900
O5—C51	1.4279 (15)	C17—C18	1.5146 (16)
O11—C11	1.2438 (14)	C17—H17A	0.9900
O18—C18	1.4220 (14)	C17—H17B	0.9900
O18—C181	1.4302 (15)	C18—H18	1.0000
O19—C18	1.4085 (14)	C31—H31A	0.9800
O19—C110	1.4319 (15)	C31—H31B	0.9800
N12—C11	1.3390 (15)	C31—H31C	0.9800
N12—C13	1.4602 (14)	C41—H41A	0.9800
N12—H12	0.856 (16)	C41—H41B	0.9800
C1—C2	1.3932 (16)	C41—H41C	0.9800
C1—C6	1.3938 (16)	C51—H51A	0.9800
C1—C11	1.5024 (15)	C51—H51B	0.9800
C2—C3	1.3901 (16)	C51—H51C	0.9800
C2—H2	0.9500	C110—C111	1.5023 (18)
C3—C4	1.3986 (17)	C110—H11A	0.9900
C4—C5	1.3985 (17)	C110—H11B	0.9900
C5—C6	1.3909 (16)	C111—H11C	0.9800
C6—H6	0.9500	C111—H11D	0.9800
C13—C14	1.5268 (16)	C111—H11E	0.9800
C13—H13A	0.9900	C181—C182	1.507 (2)
C13—H13B	0.9900	C181—H18A	0.9900
C14—C15	1.5284 (15)	C181—H18B	0.9900
C14—H14A	0.9900	C182—H18C	0.9800
C14—H14B	0.9900	C182—H18D	0.9800
C15—C16	1.5214 (16)	C182—H18E	0.9800
			
C3—O3—C31	117.13 (9)	C18—C17—H17A	108.9
C4—O4—C41	116.31 (9)	C16—C17—H17A	108.9
C5—O5—C51	117.14 (9)	C18—C17—H17B	108.9
C18—O18—C181	115.25 (9)	C16—C17—H17B	108.9
C18—O19—C110	112.74 (9)	H17A—C17—H17B	107.7
C11—N12—C13	123.36 (10)	O19—C18—O18	111.37 (9)
C11—N12—H12	118.9 (10)	O19—C18—C17	107.31 (9)
C13—N12—H12	117.7 (10)	O18—C18—C17	114.24 (9)
C2—C1—C6	120.44 (10)	O19—C18—H18	107.9
C2—C1—C11	116.72 (10)	O18—C18—H18	107.9
C6—C1—C11	122.80 (10)	C17—C18—H18	107.9
C3—C2—C1	119.90 (10)	O3—C31—H31A	109.5
C3—C2—H2	120.0	O3—C31—H31B	109.5
C1—C2—H2	120.0	H31A—C31—H31B	109.5
O3—C3—C2	124.15 (10)	O3—C31—H31C	109.5
O3—C3—C4	115.58 (10)	H31A—C31—H31C	109.5
C2—C3—C4	120.26 (10)	H31B—C31—H31C	109.5
O4—C4—C5	122.78 (11)	O4—C41—H41A	109.5
O4—C4—C3	117.74 (10)	O4—C41—H41B	109.5
C5—C4—C3	119.25 (10)	H41A—C41—H41B	109.5
O5—C5—C6	123.85 (11)	O4—C41—H41C	109.5
O5—C5—C4	115.44 (10)	H41A—C41—H41C	109.5
C6—C5—C4	120.70 (11)	H41B—C41—H41C	109.5
C5—C6—C1	119.40 (10)	O5—C51—H51A	109.5
C5—C6—H6	120.3	O5—C51—H51B	109.5
C1—C6—H6	120.3	H51A—C51—H51B	109.5
O11—C11—N12	122.70 (10)	O5—C51—H51C	109.5
O11—C11—C1	120.36 (10)	H51A—C51—H51C	109.5
N12—C11—C1	116.88 (10)	H51B—C51—H51C	109.5
N12—C13—C14	110.54 (9)	O19—C110—C111	107.35 (11)
N12—C13—H13A	109.5	O19—C110—H11A	110.2
C14—C13—H13A	109.5	C111—C110—H11A	110.2
N12—C13—H13B	109.5	O19—C110—H11B	110.2
C14—C13—H13B	109.5	C111—C110—H11B	110.2
H13A—C13—H13B	108.1	H11A—C110—H11B	108.5
C13—C14—C15	113.47 (9)	C110—C111—H11C	109.5
C13—C14—H14A	108.9	C110—C111—H11D	109.5
C15—C14—H14A	108.9	H11C—C111—H11D	109.5
C13—C14—H14B	108.9	C110—C111—H11E	109.5
C15—C14—H14B	108.9	H11C—C111—H11E	109.5
H14A—C14—H14B	107.7	H11D—C111—H11E	109.5
C16—C15—C14	114.66 (9)	O18—C181—C182	108.10 (11)
C16—C15—H15A	108.6	O18—C181—H18A	110.1
C14—C15—H15A	108.6	C182—C181—H18A	110.1
C16—C15—H15B	108.6	O18—C181—H18B	110.1
C14—C15—H15B	108.6	C182—C181—H18B	110.1
H15A—C15—H15B	107.6	H18A—C181—H18B	108.4
C15—C16—C17	111.13 (9)	C181—C182—H18C	109.5
C15—C16—H16A	109.4	C181—C182—H18D	109.5
C17—C16—H16A	109.4	H18C—C182—H18D	109.5
C15—C16—H16B	109.4	C181—C182—H18E	109.5
C17—C16—H16B	109.4	H18C—C182—H18E	109.5
H16A—C16—H16B	108.0	H18D—C182—H18E	109.5
C18—C17—C16	113.51 (10)		
			
C6—C1—C2—C3	1.44 (16)	C11—C1—C6—C5	176.84 (10)
C11—C1—C2—C3	−176.27 (10)	C13—N12—C11—O11	7.15 (17)
C31—O3—C3—C2	9.59 (16)	C13—N12—C11—C1	−170.25 (10)
C31—O3—C3—C4	−171.49 (10)	C2—C1—C11—O11	−18.88 (15)
C1—C2—C3—O3	178.65 (10)	C6—C1—C11—O11	163.47 (10)
C1—C2—C3—C4	−0.23 (16)	C2—C1—C11—N12	158.58 (10)
C41—O4—C4—C5	61.51 (15)	C6—C1—C11—N12	−19.07 (15)
C41—O4—C4—C3	−124.05 (12)	C11—N12—C13—C14	114.65 (12)
O3—C3—C4—O4	4.74 (15)	N12—C13—C14—C15	175.72 (9)
C2—C3—C4—O4	−176.30 (10)	C13—C14—C15—C16	67.27 (13)
O3—C3—C4—C5	179.37 (10)	C14—C15—C16—C17	175.71 (10)
C2—C3—C4—C5	−1.66 (17)	C15—C16—C17—C18	−177.76 (10)
C51—O5—C5—C6	9.66 (17)	C110—O19—C18—O18	67.61 (12)
C51—O5—C5—C4	−171.35 (11)	C110—O19—C18—C17	−166.69 (10)
O4—C4—C5—O5	−2.29 (16)	C181—O18—C18—O19	62.01 (12)
C3—C4—C5—O5	−176.64 (10)	C181—O18—C18—C17	−59.76 (13)
O4—C4—C5—C6	176.74 (10)	C16—C17—C18—O19	178.26 (9)
C3—C4—C5—C6	2.39 (17)	C16—C17—C18—O18	−57.77 (13)
O5—C5—C6—C1	177.74 (10)	C18—O19—C110—C111	−179.38 (10)
C4—C5—C6—C1	−1.20 (17)	C18—O18—C181—C182	−160.85 (10)
C2—C1—C6—C5	−0.72 (16)		

### (3) N-(6,6-Diethoxyhexyl)-3,4,5-trimethoxybenzamide Hydrogen-bond geometry (Å, º)

**Table:**

*D*—H···*A*	*D*—H	H···*A*	*D*···*A*	*D*—H···*A*
N12—H12···O11^i^	0.856 (16)	2.169 (16)	2.9890 (13)	160.2 (14)
C6—H6···O11^i^	0.95	2.34	3.2549 (14)	162
C15—H15*B*···O18^ii^	0.99	2.49	3.4239 (14)	157

Symmetry codes: (i) *x*, −*y*+3/2, *z*−1/2; (ii) *x*, −*y*+1/2, *z*−1/2.

## References

[bb1] Agilent (2014). *CrysAlis PRO*. Agilent Technologies UK Ltd, Yarnton, England.

[bb2] Benfeito, S., Oliveira, C., Soares, P., Fernandes, C., Silva, T., Teixeira, J. & Borges, F. (2013). *Mitochondrion*, **13**, 427–435. 10.1016/j.mito.2012.12.002 23246773

[bb3] Coles, S. J. & Gale, P. A. (2012). *Chem. Sci.* **3**, 683–689.

[bb4] Dillen, J., Woldu, M. G. & Koch, K. R. (2006). *Acta Cryst.* E**62**, o5225–o5227.

[bb5] Dolomanov, O. V., Bourhis, L. J., Gildea, R. J., Howard, J. A. K. & Puschmann, H. (2009). *J. Appl. Cryst.* **42**, 339–341.

[bb6] Garrido, J. & Borges, F. (2013). *Food. Res. Int.* **54**, 1844–1858.

[bb7] Groom, C. R., Bruno, I. J., Lightfoot, M. P. & Ward, S. C. (2016). *Acta Cryst* B**72**, 171–179. 10.1107/S2052520616003954 PMC4822653 27048719

[bb8] Hübschle, C. B., Sheldrick, G. M. & Dittrich, B. (2011). *J. Appl. Cryst.* **44**, 1281–1284. 10.1107/S0021889811043202 PMC3246833 22477785

[bb9] Macrae, C. F., Edgington, P. R., McCabe, P., Pidcock, E., Shields, G. P., Taylor, R., Towler, M. & van de Streek, J. (2006). *J. Appl. Cryst.* **39**, 453–457.

[bb10] McArdle, P., Gilligan, K., Cunningham, D., Dark, R. & Mahon, M. (2004). *CrystEngComm*, **6**, 303–309.

[bb11] Oszlányi, G. & Sütő, A. (2004). *Acta Cryst.* A**60**, 134–141. 10.1107/S0108767303027569 14966324

[bb12] Rigaku (2012). *CrystalClear-SM Expert*. Rigaku Corporation, Tokyo, Japan.

[bb13] Rohl, A. L., Moret, M., Kaminsky, W., Claborn, K., McKinnon, J. J. & Kahr, B. (2008). *Cryst. Growth Des.* **8**, 4517–4525.

[bb14] Roleira, F. M. F., Tavares-da-Silva, E. J., Varela, C. L., Costa, S. C., Silva, T., Garrido, J. & Borges, F. (2015). *Food Chem.* **183**, 235–258. 10.1016/j.foodchem.2015.03.039 25863633

[bb15] Sheldrick, G. M. (2015*a*). *Acta Cryst.* A**71**, 3–8.

[bb16] Sheldrick, G. M. (2015*b*). *Acta Cryst.* C**71**, 3–8.

[bb17] Spek, A. L. (2009). *Acta Cryst.* D**65**, 148–155. 10.1107/S090744490804362X PMC2631630 19171970

[bb18] Sun, Y.-F., Sun, X.-Z., Li, J.-K. & Zheng, Z.-B. (2007). *Acta Cryst.* E**63**, o2180–o2181.

[bb19] Teixeira, J., Silva, T., Andrade, P. B. & Borges, F. (2013). *Curr. Med. Chem.* **20**, 2939–2952. 10.2174/1871523011320240001 23409717

[bb20] Tsuzuki, S., Houjou, H., Nagawa, & Hiratani, K. (2002). *J. Chem. Soc. Perkin Trans. 2*, pp. 1271–1273.

[bb21] Wolff, S. K., Grimwood, D. J., McKinnon, J. J., Turner, M. J., Jayatilaka, D. & Spackman, M. A. (2012). *Crystal Explorer*. The University of Western Australia.

