# Supplementary material for: Crystal structures of three 3,4,5-tri­meth­oxy­benzamide-based derivatives
Source: Acta Crystallogr E Crystallogr Commun. 2016 Apr 15;72(Pt 5):675–82. doi: 10.1107/S2056989016005958 (PMC4908538; doi:10.1107/S2056989016005958)

# Search Overview

***Search:*** search2

***Date/Time done:*** Wed Mar 16 11:04:25 2016

***Database(s):*** CSD version 5.37 updates (Nov 2015)
CSD version 5.37 (November 2015)
CSD version 5.37 (November 2015)
CSD version 5.37 updates (Feb 2016)

***Restriction Info:*** No refcode restrictions applied

***Filters:*** 3D coordinates determined
No errors
No ions
Only Organics
Not disordered
Not polymeric
No powder structures

***Percentage Completed:*** 100%

***Number of Hits:*** 19

***Single query used. Search found structures that:***

match
**Query 1**

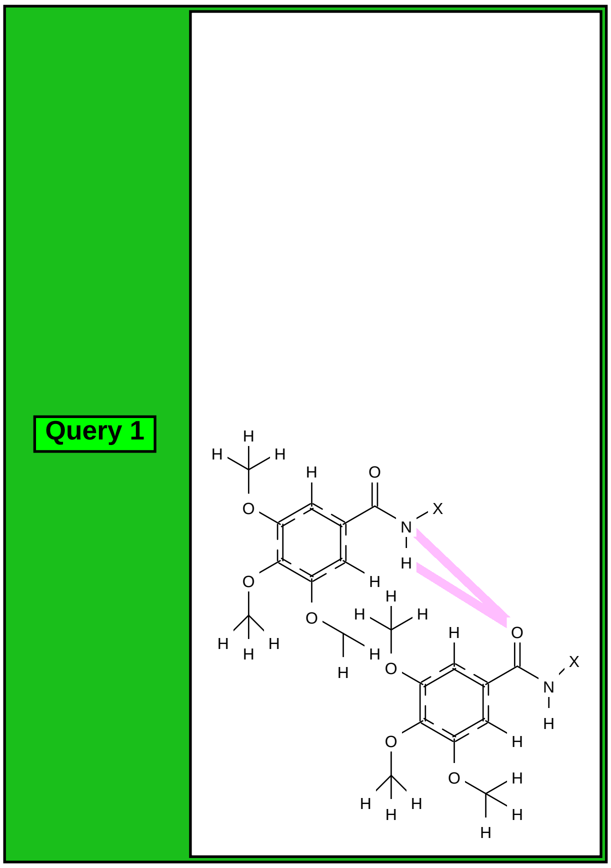

# Search: search2 (Wed Mar 16 11:04:25 2016): Hits 1-4

## DUJMIF

***Reference:*** A.Saeed, M.Arshad, R.A.Khera, M.Bolte (2009) *Acta Crystallogr.,Sect.E:Struct.Rep.Online* ,**65,**o3234

***Formula:*** $C_{16} H_{23} N_1 O_4$

***Compound Name:*** N-Cyclohexyl-3,4,5-trimethoxybenzamide

| | | | | | | | |
|---|---|---|---|---|---|---|---|
| ***Space Group:*** | P21/c | ***Cell:*** | ***a*** 23.454(1) | ***b*** 5.215(0) | ***c*** 12.456(1) |
| ***Space Group No.:*** | 14 | ***(Å,°)*** | α 90.00 | β 92.89(0) | γ 90.00 |

***R-Factor (%):*** 4.00 ***Temperature(K):*** 173 ***Density(g/cm$^3$):*** 1.281

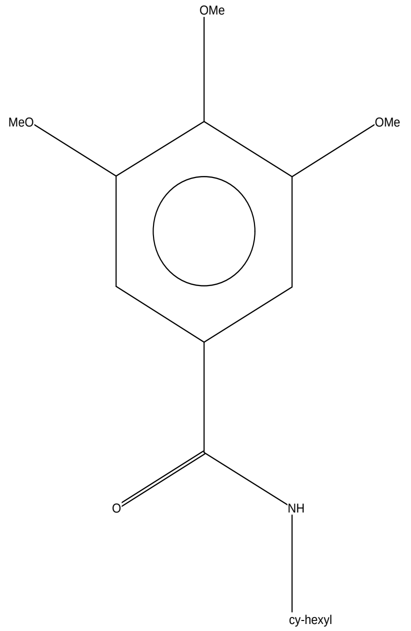


***Parameters***

| *Fragment 1* | |
|---|---|
| **CCNX (T)** | -175.930 |
| **NTOO (D)** | 3.026 |
| **HTOO (D)** | 2.152 |
| **NTOH (D)** | 0.917 |
| **C2N2 (T)** | 147.364 |
| **C6N2 (T)** | -34.942 |

## FETYUZ

***Reference:*** Xun Li, Wen-Fang Xu, Ji-Feng Wu, Jun-Li Wang, Yu-Mei Yuan (2005) *Acta Crystallogr.,Sect.E:Struct.Rep.Online* ,**61,** o349

***Formula:*** $C_{18} H_{24} N_2 O_9$

***Compound Name:*** (4S)-4-(Methoxycarbonylmethylaminocarbonyl)-4-(3,4,5-trimethoxybenzamido)butanoic acid

| | | | | | |
|---|---|---|---|---|---|
| ***Space Group:*** | P21/c | ***Cell:*** | ***a*** 27.665(9) | ***b*** 5.144(1) | ***c*** 13.907(4) |
| ***Space Group No.:*** | 14 | ***(Å,°)*** | α 90.00 | β 98.40(0) | γ 90.00 |

***R-Factor (%):*** 6.06 ***Temperature(K):*** 298 ***Density(g/cm$^3$):*** 1.399

***Parameters***

| *Fragment 1* | |
|---|---|
| **CCNX (T)** | -177.170 |
| **NTOO (D)** | 3.059 |
| **HTOO (D)** | 2.249 |
| **NTOH (D)** | 0.860 |
| **C2N2 (T)** | 34.621 |
| **C6N2 (T)** | -148.360 |

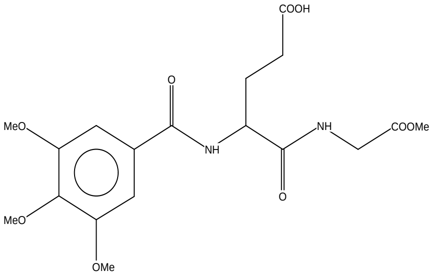


## FOWXIZ

***Reference:*** A.Saeed, U.Florke (2009) *Acta Crystallogr.,Sect.E:Struct.Rep.Online* ,**65,**o1948

***Formula:*** $C_{17} H_{19} N_1 O_5$

***Compound Name:*** 3,4,5-Trimethoxy-N-(2-methoxyphenyl)benzamide

| | | | | | |
|---|---|---|---|---|---|
| ***Space Group:*** | Pca21 | ***Cell:*** | ***a*** 7.409(2) | ***b*** 22.522(6) | ***c*** 9.681(3) |
| ***Space Group No.:*** | 29 | ***(Å,°)*** | α 90.00 | β 90.00 | γ 90.00 |

***R-Factor (%):*** 5.09 ***Temperature(K):*** 120 ***Density(g/cm$^3$):*** 1.305

***Parameters***

| *Fragment 1* | |
|---|---|
| **CCNX (T)** | 174.026 |
| **NTOO (D)** | 3.066 |
| **HTOO (D)** | 2.180 |
| **NTOH (D)** | 0.897 |
| **C2N2 (T)** | -27.099 |
| **C6N2 (T)** | 157.566 |

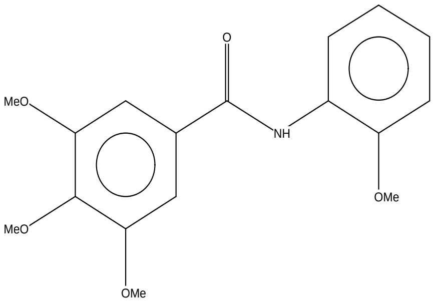


## HOZCEF

***Reference:*** A.Saeed, S.Hussain, A.Ibrar, M.Bolte (2009) *Acta Crystallogr.,Sect.E:Struct.Rep.Online* ,**65,**o1470

***Formula:*** $C_{16} H_{16} Br_1 N_1 O_4$

***Compound Name:*** N-(3-Bromophenyl)-3,4,5-trimethoxybenzamide

| | | | | | |
|---|---|---|---|---|---|
| ***Space Group:*** | Pna21 | ***Cell:*** | ***a*** 13.309(0) | ***b*** 4.995(0) | ***c*** 23.406(1) |
| ***Space Group No.:*** | 33 | ***(Å,°)*** | α 90.00 | β 90.00 | γ 90.00 |

***R-Factor (%):*** 3.29 ***Temperature(K):*** 173 ***Density(g/cm$^3$):*** 1.563

***Parameters***

| *Fragment 1* | |
|---|---|
| **CCNX (T)** | 178.313 |
| **NTOO (D)** | 2.821 |
| **HTOO (D)** | 2.058 |
| **NTOH (D)** | 0.854 |
| **C2N2 (T)** | -146.792 |
| **C6N2 (T)** | 34.628 |

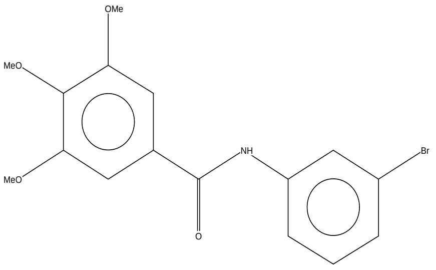

# Search: search2 (Wed Mar 16 11:04:25 2016): Hits 5-8

## MODSOO

***Reference:*** A.Saeed, R.A.Khera, M.Batool, U.Shaheen, U.Florke (2008) *Acta Crystallogr.,Sect.E:Struct.Rep.Online* ,**64,**o1625

***Formula:*** $C_{16}\ H_{16}\ Cl_1\ N_1\ O_4$

***Compound Name:*** N-(4-Chlorophenyl)-3,4,5-trimethoxybenzamide

| | | | | | | | | |
|---|---|---|---|---|---|---|---|---|
| ***Space Group:*** | Cc | ***Cell:*** | ***a*** | 9.487(2) | ***b*** | 25.666(6) | ***c*** | 6.978(1) |
| ***Space Group No.:*** | 9 | ***(Å,°)*** | α | 90.00 | β | 112.34(0) | γ | 90.00 |

***R-Factor (%):*** 4.50 ***Temperature(K):*** 120 ***Density(g/cm$^3$):*** 1.360

***Parameters***

*Fragment 1*

| | | |
|---|---|---|
| **CCNX** | **(T)** | -178.492 |
| **NTOO** | **(D)** | 2.879 |
| **HTOO** | **(D)** | 2.184 |
| **NTOH** | **(D)** | 0.880 |
| **C2N2** | **(T)** | -31.101 |
| **C6N2** | **(T)** | 150.813 |

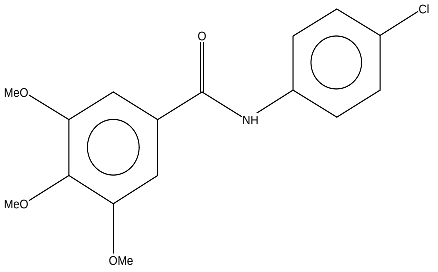


## MODXAF

***Reference:*** Dao-Hang He, Yong-Chuang Zhu, Zhuo-Ru Yang (2008) *Acta Crystallogr.,Sect.E:Struct.Rep.Online* ,**64,**o1648

***Formula:*** $C_{17}\ H_{17}\ F_1\ N_2\ O_4$

***Compound Name:*** N'-(4-Fluorobenzylidene)-3,4,5-trimethoxybenzohydrazide

| | | | | | | | | |
|---|---|---|---|---|---|---|---|---|
| ***Space Group:*** | P21/c | ***Cell:*** | ***a*** | 7.919(0) | ***b*** | 26.250(1) | ***c*** | 8.127(0) |
| ***Space Group No.:*** | 14 | ***(Å,°)*** | α | 90.00 | β | 105.55(0) | γ | 90.00 |

***R-Factor (%):*** 3.70 ***Temperature(K):*** 173 ***Density(g/cm$^3$):*** 1.356

***Parameters***

*Fragment 1*

| | | |
|---|---|---|
| **CCNX** | **(T)** | 177.116 |
| **NTOO** | **(D)** | 2.824 |
| **HTOO** | **(D)** | 1.961 |
| **NTOH** | **(D)** | 0.880 |
| **C2N2** | **(T)** | 142.263 |
| **C6N2** | **(T)** | -35.627 |

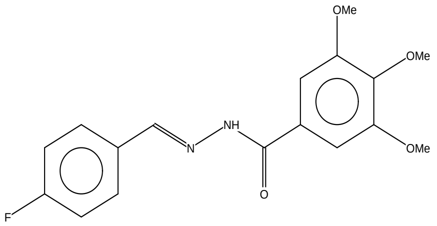


## NICHOY

***Reference:*** A.Saeed, J.Simpson (2013) *J.Chem.Cryst.* ,**43,**51

***Formula:*** $C_{17}\ H_{19}\ N_1\ O_4$

***Compound Name:*** 3,4,5-Trimethoxy-N-(4-methylphenyl)benzamide

***Synonym:*** 3,4,5-trimethoxy-N-p-tolylbenzamide

| | | | | | | | | |
|---|---|---|---|---|---|---|---|---|
| ***Space Group:*** | P21 | ***Cell:*** | ***a*** | 5.107(0) | ***b*** | 13.915(1) | ***c*** | 11.205(1) |
| ***Space Group No.:*** | 4 | ***(Å,°)*** | α | 90.00 | β | 103.12(0) | γ | 90.00 |

***R-Factor (%):*** 3.77 ***Temperature(K):*** 90 ***Density(g/cm$^3$):*** 1.291

***Parameters***

*Fragment 1*

| | | |
|---|---|---|
| **CCNX** | **(T)** | -178.908 |
| **NTOO** | **(D)** | 2.945 |
| **HTOO** | **(D)** | 2.061 |
| **NTOH** | **(D)** | 0.910 |
| **C2N2** | **(T)** | 148.362 |
| **C6N2** | **(T)** | -34.473 |

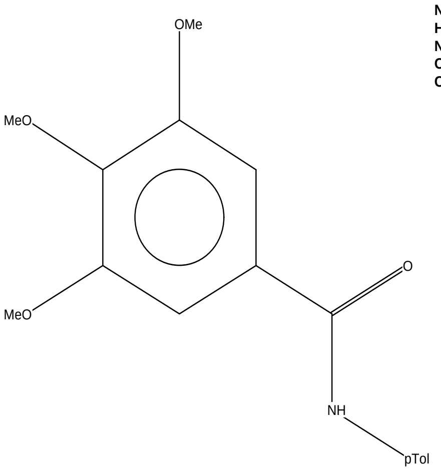


## NUQLIV

***Reference:*** H.Choi, B.H.Han, T.Lee, S.K.Kang, C.K.Sung (2010) *Acta Crystallogr.,Sect.E:Struct.Rep.Online* ,**66,**o1142

***Formula:*** $C_{16}\ H_{15}\ F_2\ N_1\ O_4$

***Compound Name:*** N-(3,4-Difluorophenyl)-3,4,5-trimethoxybenzamide

| | | | | | | | | |
|---|---|---|---|---|---|---|---|---|
| ***Space Group:*** | P21/n | ***Cell:*** | ***a*** | 5.003(0) | ***b*** | 8.899(0) | ***c*** | 32.726(2) |
| ***Space Group No.:*** | 14 | ***(Å,°)*** | α | 90.00 | β | 93.90(0) | γ | 90.00 |

***R-Factor (%):*** 6.51 ***Temperature(K):*** 174 ***Density(g/cm$^3$):*** 1.477

***Parameters***

*Fragment 1*

| | | |
|---|---|---|
| **CCNX** | **(T)** | -175.913 |
| **NTOO** | **(D)** | 2.872 |
| **HTOO** | **(D)** | 2.020 |
| **NTOH** | **(D)** | 0.926 |
| **C2N2** | **(T)** | 32.870 |
| **C6N2** | **(T)** | -150.304 |

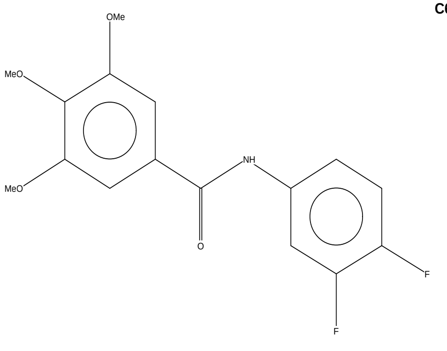

# Search: search2 (Wed Mar 16 11:04:25 2016): Hits 9-12

## PAVMAC

***Reference:*** Wen Gu, Chao Qiao (2012) *Acta Crystallogr.,Sect.E:Struct.Rep.Online* ,**68,**o1658

***Formula:*** $C_{16} H_{16} Br_1 N_1 O_4$

***Compound Name:*** N-(4-Bromophenyl)-3,4,5-trimethoxybenzamide

| | | | | | |
|---|---|---|---|---|---|
| ***Space Group:*** | Cc | ***Cell:*** | ***a*** 9.586(1) | ***b*** 26.010(5) | ***c*** 7.139(1) |
| ***Space Group No.:*** | 9 | ***(Å,°)*** | α 90.00 | β 112.04(3) | γ 90.00 |

***R-Factor (%):*** 4.47 ***Temperature(K):*** 293 ***Density(g/cm³):*** 1.474

***Parameters***

*Fragment 1*

| | |
|---|---|
| **CCNX (T)** | 175.764 |
| **NTOO (D)** | 2.908 |
| **HTOO (D)** | 2.194 |
| **NTOH (D)** | 0.860 |
| **C2N2 (T)** | -146.396 |
| **C6N2 (T)** | 36.434 |

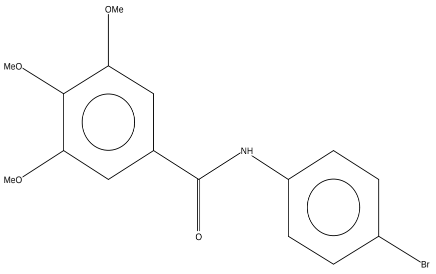


## PIXJIQ

***Reference:*** L.Bonardi, H.Kanaan, F.Camerel, P.Jolinat, P.Retailleau, R.Ziessel (2008) *Adv.Funct.Mater.* ,**18,**401

***Formula:*** $C_{33} H_{38} B_1 F_2 N_3 O_4$

***Compound Name:*** 2,6-Diethyl-4,4-difluoro-1,3,5,7-tetramethyl-8-(4-(3,4,5-trimethoxybenzoylamino)phenyl)-4,4a-dihydro-3a,4a-diaza-4-bora-s-indacene

| | | | | | |
|---|---|---|---|---|---|
| ***Space Group:*** | P21/c | ***Cell:*** | ***a*** 15.036(1) | ***b*** 21.640(2) | ***c*** 10.101(1) |
| ***Space Group No.:*** | 14 | ***(Å,°)*** | α 90.00 | β 106.84(0) | γ 90.00 |

***R-Factor (%):*** 5.24 ***Temperature(K):*** 293 ***Density(g/cm³):*** 1.245

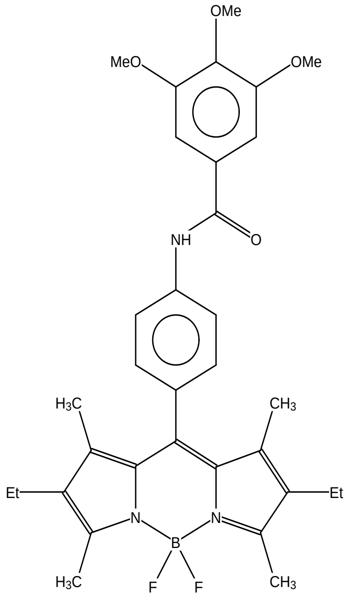


***Parameters***

*Fragment 1*

| | |
|---|---|
| **CCNX (T)** | -171.301 |
| **NTOO (D)** | 2.998 |
| **HTOO (D)** | 2.243 |
| **NTOH (D)** | 0.815 |
| **C2N2 (T)** | -29.729 |
| **C6N2 (T)** | 148.584 |

## RIGCOA

***Reference:*** M.Zareef, R.Iqbal, G.Qadeer, M.Arfan, Wai-Yeung Wong (2007) *Acta Crystallogr.,Sect.E:Struct.Rep.Online* ,**63,**o3052

***Formula:*** $C_{17} H_{17} F_1 N_2 O_5 ,H_2 O_1$

***Compound Name:*** N'-(4-Fluorobenzoyl)-3,4,5-trimethoxybenzohydrazide monohydrate

| | | | | | |
|---|---|---|---|---|---|
| ***Space Group:*** | P21/c | ***Cell:*** | ***a*** 8.385(0) | ***b*** 13.269(0) | ***c*** 31.833(2) |
| ***Space Group No.:*** | 14 | ***(Å,°)*** | α 90.00 | β 94.62(0) | γ 90.00 |

***R-Factor (%):*** 4.81 ***Temperature(K):*** 294 ***Density(g/cm³):*** 1.379

***Parameters***

*Fragment 1*

| | |
|---|---|
| **CCNX (T)** | -178.613 |
| **NTOO (D)** | 2.814 |
| **HTOO (D)** | 2.064 |
| **NTOH (D)** | 0.859 |
| **C2N2 (T)** | 30.758 |
| **C6N2 (T)** | -152.478 |

*Fragment 2*

| | |
|---|---|
| **CCNX (T)** | 169.244 |
| **NTOO (D)** | 2.814 |
| **HTOO (D)** | 2.000 |
| **NTOH (D)** | 0.860 |
| **C2N2 (T)** | -34.219 |
| **C6N2 (T)** | 150.568 |

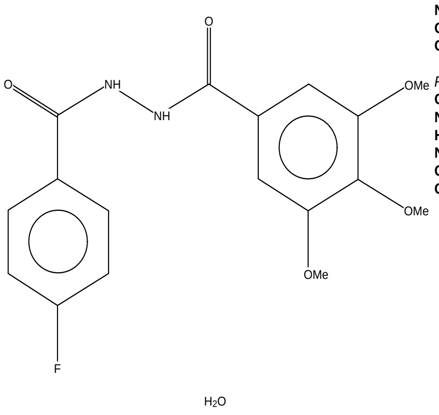


## SUJFUA

***Reference:*** T.K.Achar, P.Mal (2014) *J.Org.Chem.* ,**80,**666

***Formula:*** $C_{17} H_{19} N_1 O_4$

***Compound Name:*** N-Benzyl-3,4,5-trimethoxybenzamide

| | | | | | |
|---|---|---|---|---|---|
| ***Space Group:*** | P21/c | ***Cell:*** | ***a*** 23.000(0) | ***b*** 5.091(0) | ***c*** 12.942(0) |
| ***Space Group No.:*** | 14 | ***(Å,°)*** | α 90.00 | β 95.10(0) | γ 90.00 |

***R-Factor (%):*** 3.99 ***Temperature(K):*** 298 ***Density(g/cm³):*** 1.326

***Parameters***

*Fragment 1*

| | |
|---|---|
| **CCNX (T)** | 172.235 |
| **NTOO (D)** | 2.966 |
| **HTOO (D)** | 2.134 |
| **NTOH (D)** | 0.839 |
| **C2N2 (T)** | -41.227 |
| **C6N2 (T)** | 144.461 |

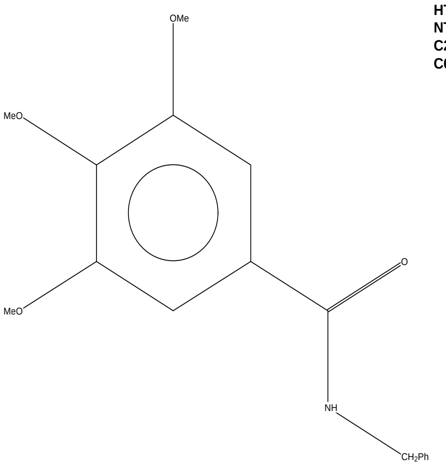

# Search: search2 (Wed Mar 16 11:04:25 2016): Hits 13-15

## VOJJOU

***Reference:*** Zhen-dong Zhao, Yu-min Wang, Yu-xiang Chen, Liang-wu Bi (2008) *Acta Crystallogr.,Sect.E:Struct.Rep.Online* ,**64,** o2408

***Formula:*** $C_{17} H_{17} Br_1 N_2 O_5$

***Compound Name:*** (E)-N'-(5-bromo-2-hydroxybenzylidene)-3,4,5-trimethoxybenzohydrazide

| | | | | | | | | |
|---|---|---|---|---|---|---|---|---|
| ***Space Group:*** | P21/c | ***Cell:*** | ***a*** | 11.416(1) | ***b*** | 16.279(3) | ***c*** | 9.374(1) |
| ***Space Group No.:*** | 14 | ***(Å,°)*** | α | 90.00 | β | 100.21(0) | γ | 90.00 |

***R-Factor (%):*** 3.69 ***Temperature(K):*** 273 ***Density(g/cm³):*** 1.586

***Parameters***

*Fragment 1*

| | |
|---|---|
| **CCNX (T)** | -179.423 |
| **NTOO (D)** | 2.888 |
| **HTOO (D)** | 2.137 |
| **NTOH (D)** | 0.860 |
| **C2N2 (T)** | 147.850 |
| **C6N2 (T)** | -33.045 |

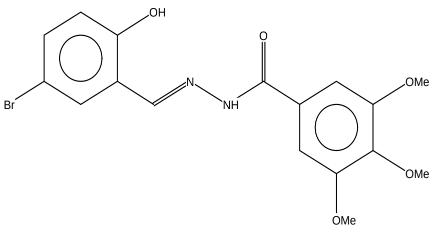


## VOJKEL

***Reference:*** Yu-Min Wang, Zhen-Dong Zhao, Yu-Xiang Chen, Liang-Wu Bi (2008) *Acta Crystallogr.,Sect.E:Struct.Rep.Online* ,**64,** o2459

***Formula:*** $C_{17} H_{17} Cl_1 N_2 O_4$

***Compound Name:*** (E)-N'-(4-chlorobenzylidene)-3,4,5-trimethoxybenzohydrazide

| | | | | | | | | |
|---|---|---|---|---|---|---|---|---|
| ***Space Group:*** | P-1 | ***Cell:*** | ***a*** | 5.119(2) | ***b*** | 8.210(4) | ***c*** | 20.276(9) |
| ***Space Group No.:*** | 2 | ***(Å,°)*** | α | 101.06(0) | β | 92.36(0) | γ | 101.46(0) |

***R-Factor (%):*** 4.61 ***Temperature(K):*** 273 ***Density(g/cm³):*** 1.418

***Parameters***

*Fragment 1*

| | |
|---|---|
| **CCNX (T)** | 174.854 |
| **NTOO (D)** | 2.943 |
| **HTOO (D)** | 2.183 |
| **NTOH (D)** | 0.860 |
| **C2N2 (T)** | -145.945 |
| **C6N2 (T)** | 37.920 |

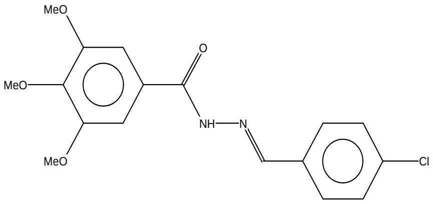


## JACHUT

***Reference:*** You-Yue Han (2013) *J.Chil.Chem.Soc.* ,**58,**1858

***Formula:*** $C_{17} H_{16} Cl_2 N_2 O_4$

***Compound Name:*** N'-(2,4-dichlorobenzylidene)-3,4,5-trimethoxybenzohydrazide

| | | | | | | | | |
|---|---|---|---|---|---|---|---|---|
| ***Space Group:*** | P21/c | ***Cell:*** | ***a*** | 17.127(3) | ***b*** | 13.326(3) | ***c*** | 7.984(1) |
| ***Space Group No.:*** | 14 | ***(Å,°)*** | α | 90.00 | β | 99.69(0) | γ | 90.00 |

***R-Factor (%):*** 4.89 ***Temperature(K):*** 298 ***Density(g/cm³):*** 1.417

***Parameters***

*Fragment 1*

| | |
|---|---|
| **CCNX (T)** | -177.783 |
| **NTOO (D)** | 2.906 |
| **HTOO (D)** | 2.051 |
| **NTOH (D)** | 0.892 |
| **C2N2 (T)** | -38.862 |
| **C6N2 (T)** | 138.900 |

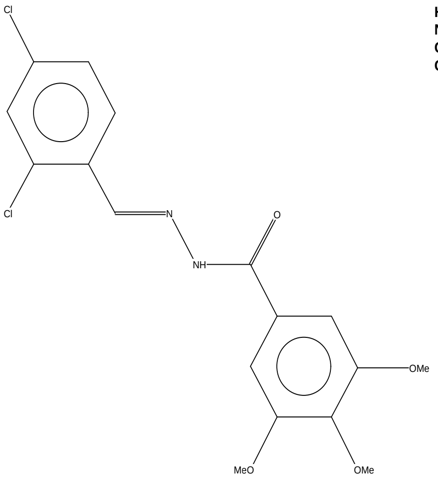

Supplement: Supplementary file 8 [file e-72-00675-sup8.pdf]
