# Supplementary material for: Crystal structures of three 3,4,5-tri­meth­oxy­benzamide-based derivatives
Source: Acta Crystallogr E Crystallogr Commun. 2016 Apr 15;72(Pt 5):675–82. doi: 10.1107/S2056989016005958 (PMC4908538; doi:10.1107/S2056989016005958)

Search Overview

Search:	search3
Date/Time done:	Wed Mar 16 10:48:50 2016
Database(s):	CSD version 5.37 updates (Nov 2015)<br>CSD version 5.37 (November 2015)<br>CSD version 5.37 (November 2015)<br>CSD version 5.37 updates (Feb 2016)
Restriction Info:	No refcode restrictions applied
Filters:	3D coordinates determined<br>No errors<br>No ions<br>Only Organics<br>Not disordered<br>Not polymeric<br>No powder structures
Percentage Completed:	100%
Number of Hits:	37

Single query used. Search found structures that:

match

Query 1

Query 1

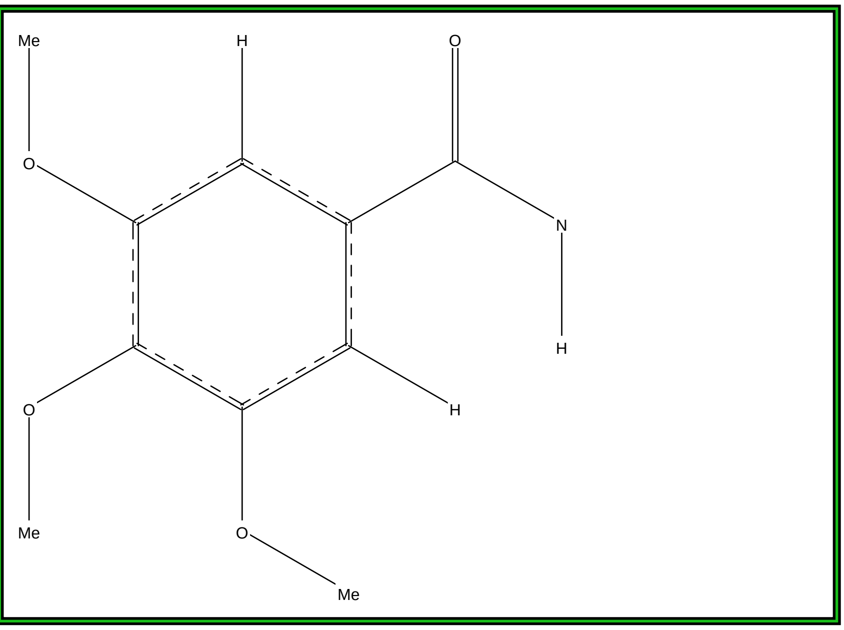

Search: search3 (Wed Mar 16 10:48:50 2016): Hits 1-4

DUJMIF

Reference: A.Saeed, M.Arshad, R.A.Khera, M.Bolte (2009) *Acta Crystallogr.,Sect.E:Struct.Rep.Online* ,**65,**o3234

Formula: $C_{16} H_{23} N_1 O_4$

Compound Name: N-Cyclohexyl-3,4,5-trimethoxybenzamide

Space Group:	P21/c	***Cell:***	***a***	23.454(1)	***b***	5.215(0)	***c***	12.456(1)
Space Group No.:	14	***(Å,°)***	α	90.00	β	92.89(0)	γ	90.00

R-Factor (%): 4.00 ***Temperature(K):*** 173 ***Density(g/cm³):*** 1.281

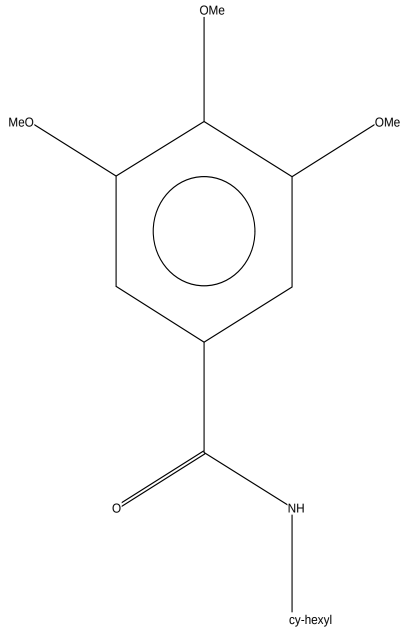

Parameters

Fragment 1

**TOR3**	**(T)**	-1.534
**TOR4**	**(T)**	-108.198
**TOR5**	**(T)**	0.725
**C6N2**	**(T)**	147.364
**C2N2**	**(T)**	-34.942

FAQPOE

Reference: A.Saeed, A.Mumtaz, H.Ishida (2011) *J.Sulfur Chem.* ,**32,**45

Formula: $C_{17} H_{19} N_3 O_6 S_2,2(H_2 O_1)$

Compound Name: 3,4,5-Trimethoxy-N-((4-sulfamoylphenyl)carbamothioyl)benzamide dihydrate

Space Group:	P-1	***Cell:***	***a***	7.416(0)	***b***	8.752(0)	***c***	15.950(1)
Space Group No.:	2	***(Å,°)***	α	88.75(0)	β	80.47(0)	γ	88.04(0)

R-Factor (%): 3.38 ***Temperature(K):*** 223 ***Density(g/cm³):*** 1.502

Parameters

Fragment 1

**TOR3**	**(T)**	-4.391
**TOR4**	**(T)**	-75.879
**TOR5**	**(T)**	-2.838
**C6N2**	**(T)**	-10.992
**C2N2**	**(T)**	170.525

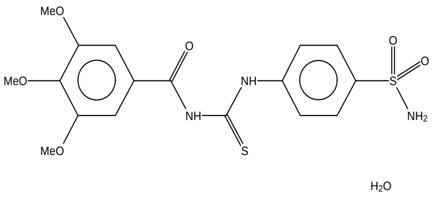

FETYUZ

Reference: Xun Li, Wen-Fang Xu, Ji-Feng Wu, Jun-Li Wang, Yu-Mei Yuan (2005) *Acta Crystallogr.,Sect.E:Struct.Rep.Online* ,**61,** o349

Formula: $C_{18} H_{24} N_2 O_9$

Compound Name: (4S)-4-(Methoxycarbonylmethylaminocarbonyl)-4-(3,4,5-trimethoxybenzamido)butanoic acid

Space Group:	P21/c	***Cell:***	***a***	27.665(9)	***b***	5.144(1)	***c***	13.907(4)
Space Group No.:	14	***(Å,°)***	α	90.00	β	98.40(0)	γ	90.00

R-Factor (%): 6.06 ***Temperature(K):*** 298 ***Density(g/cm³):*** 1.399

Parameters

Fragment 1

**TOR3**	**(T)**	4.334
**TOR4**	**(T)**	-95.327
**TOR5**	**(T)**	-0.193
**C6N2**	**(T)**	34.621
**C2N2**	**(T)**	-148.360

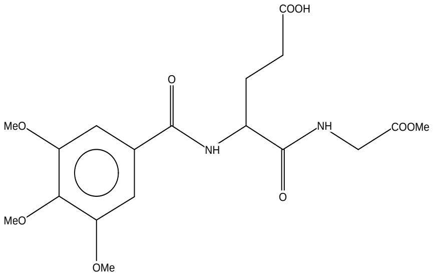

FOWXIZ

Reference: A.Saeed, U.Florke (2009) *Acta Crystallogr.,Sect.E:Struct.Rep.Online* ,**65,**o1948

Formula: $C_{17} H_{19} N_1 O_5$

Compound Name: 3,4,5-Trimethoxy-N-(2-methoxyphenyl)benzamide

Space Group:	Pca21	***Cell:***	***a***	7.409(2)	***b***	22.522(6)	***c***	9.681(3)
Space Group No.:	29	***(Å,°)***	α	90.00	β	90.00	γ	90.00

R-Factor (%): 5.09 ***Temperature(K):*** 120 ***Density(g/cm³):*** 1.305

Parameters

Fragment 1

**TOR3**	**(T)**	3.063
**TOR4**	**(T)**	103.871
**TOR5**	**(T)**	-13.314
**C6N2**	**(T)**	-27.099
**C2N2**	**(T)**	157.566

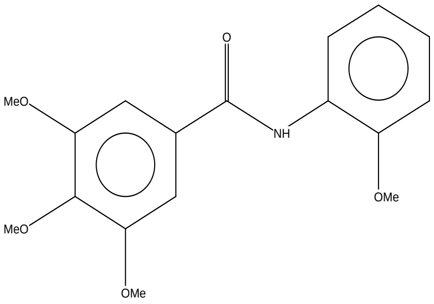

Search: search3 (Wed Mar 16 10:48:50 2016): Hits 5-8

HESLEX

Reference: J.Dillen, M.G.Woldu, K.R.Koch (2006) *Acta Crystallogr.,Sect.E:Struct.Rep.Online* ,**62,**o5225

Formula: C_{18} H_{26} N_2 O_4 S_1

Compound Name: N,N-(Heptane-2,6-diyl)-N'-(3,4,5-trimethoxybenzoyl)thiourea

Space Group: P-1
Space Group No.: 2

Cell: ***(Å,°)*** ***a*** 12.427(2) ***b*** 12.666(2) ***c*** 13.894(2) α 96.49(0) β 91.76(0) γ 118.40(0)

R-Factor (%): 4.80 ***Temperature(K):*** 173 ***Density(g/cm³):*** 1.280

Parameters

Fragment 1	
**TOR3 (T)**	-6.386
**TOR4 (T)**	-78.188
**TOR5 (T)**	-1.304
**C6N2 (T)**	-169.361
**C2N2 (T)**	13.783

Fragment 2	
**TOR3 (T)**	-10.005
**TOR4 (T)**	84.324
**TOR5 (T)**	3.142
**C6N2 (T)**	-179.322
**C2N2 (T)**	4.210

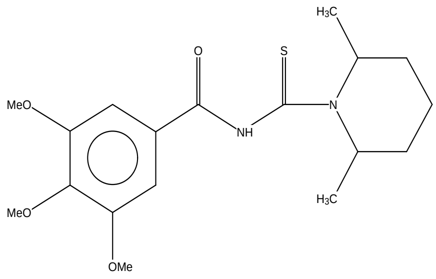

HOZCEF

Reference: A.Saeed, S.Hussain, A.Ibrar, M.Bolte (2009) *Acta Crystallogr.,Sect.E:Struct.Rep.Online* ,**65,**o1470

Formula: C_{16} H_{16} Br_1 N_1 O_4

Compound Name: N-(3-Bromophenyl)-3,4,5-trimethoxybenzamide

Space Group: Pna21
Space Group No.: 33

Cell: ***(Å,°)*** ***a*** 13.309(0) ***b*** 4.995(0) ***c*** 23.406(1) α 90.00 β 90.00 γ 90.00

R-Factor (%): 3.29 ***Temperature(K):*** 173 ***Density(g/cm³):*** 1.563

Parameters

Fragment 1	
**TOR3 (T)**	-2.525
**TOR4 (T)**	111.769
**TOR5 (T)**	-4.598
**C6N2 (T)**	-146.792
**C2N2 (T)**	34.628

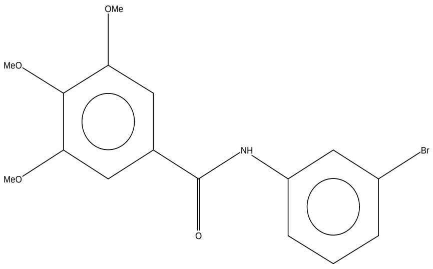

ICEGEE

Reference: A.Saeed, U.Shaheen, M.Bolte (2011) *Crystals* ,**1,**34

Formula: C_{17} H_{17} F_1 N_2 O_4 S_1

Compound Name: N-((3-Fluorophenyl)carbamothioyl)-3,4,5-trimethoxybenzamide

Space Group: P21/c
Space Group No.: 14

Cell: ***(Å,°)*** ***a*** 13.097(0) ***b*** 16.646(1) ***c*** 7.845(0) α 90.00 β 106.72(0) γ 90.00

R-Factor (%): 2.89 ***Temperature(K):*** 173 ***Density(g/cm³):*** 1.478

Parameters

Fragment 1	
**TOR3 (T)**	13.696
**TOR4 (T)**	76.195
**TOR5 (T)**	0.124
**C6N2 (T)**	-37.920
**C2N2 (T)**	145.760

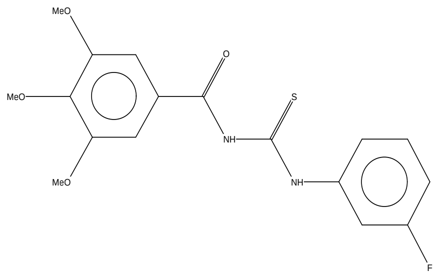

IHANOU

Reference: S.Ianelli, M.Carcelli (2001) *J.Chem.Cryst.* ,**31,**123

Formula: C_{34} H_{34} N_4 O_8

Compound Name: (E,E)-Benzil bis(3,4,5-trimethoxybenzohydrazone)

Space Group: P21/n
Space Group No.: 14

Cell: ***(Å,°)*** ***a*** 11.464(2) ***b*** 21.098(4) ***c*** 13.837(2) α 90.00 β 109.50(10) γ 90.00

R-Factor (%): 3.69 ***Temperature(K):*** 293 ***Density(g/cm³):*** 1.319

Parameters

Fragment 1	
**TOR3 (T)**	-11.722
**TOR4 (T)**	83.758
**TOR5 (T)**	7.644
**C6N2 (T)**	-163.434
**C2N2 (T)**	20.130

Fragment 2	
**TOR3 (T)**	11.481
**TOR4 (T)**	-121.689
**TOR5 (T)**	9.398
**C6N2 (T)**	158.514
**C2N2 (T)**	-22.013

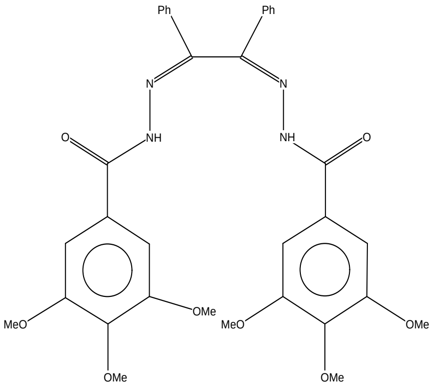

Search: search3 (Wed Mar 16 10:48:50 2016): Hits 9-12

KEVQUY

Reference: Yifeng Sun, Dongdi Zhang, Hongyin Gao, Huicheng Wang, Ruojie Tao (2006) *Anal.Sci.:X-Ray Struct.Anal.Online* ,**22,**x289

Formula: $C_{27}\ H_{26}\ N_4\ O_5$

Compound Name: N-(α-(1-Phenyl-3-methyl-5-pyrazolone-4-ylidene)benzyl)-N'-3,4,5-trimethoxybenzoylhydrazine

Space Group:	P-1	***Cell:***	***a***	9.190(1)	***b***	12.201(2)	***c***	12.500(3)
Space Group No.:	2	***(Å,°)***	α	78.32(3)	β	74.23(3)	γ	68.86(3)

R-Factor (%): 6.05 ***Temperature(K):*** 291 ***Density(g/cm³):*** 1.293

Parameters

Fragment 1

**TOR3**	**(T)**	3.062
**TOR4**	**(T)**	104.007
**TOR5**	**(T)**	-4.492
**C6N2**	**(T)**	-154.196
**C2N2**	**(T)**	22.439

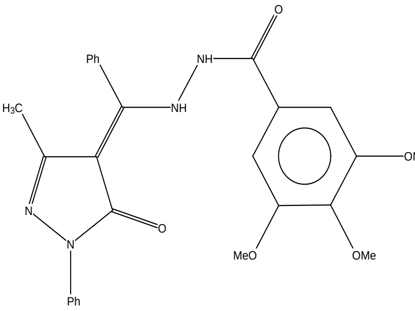

KOJCOD

Reference: Y.Ishikawa, K.Watanabe (2014) *Acta Crystallogr.,Sect.E:Struct.Rep.Online* ,**70,**o832

Formula: $C_{21}\ H_{20}\ N_2\ O_7,H_2\ O_1$

Compound Name: 3,4,5-trimethoxy-N'-((6-methoxy-4-oxo-4H-chromen-3-yl)methylene) benzohydrazide monohydrate

Space Group:	P-1	***Cell:***	***a***	7.782(3)	***b***	9.015(5)	***c***	14.991(6)
Space Group No.:	2	***(Å,°)***	α	103.17(5)	β	96.51(3)	γ	95.52(4)

R-Factor (%): 7.64 ***Temperature(K):*** 100 ***Density(g/cm³):*** 1.416

Parameters

Fragment 1

**TOR3**	**(T)**	-9.813
**TOR4**	**(T)**	-78.064
**TOR5**	**(T)**	5.334
**C6N2**	**(T)**	35.261
**C2N2**	**(T)**	-145.192

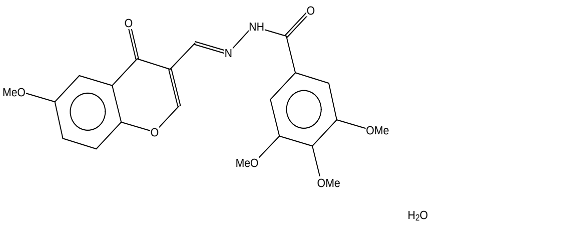

KUZSIH

Reference: C.Burgos, M.L.Izquierdo, M.S.Arias, E.Galvez, J.Sanz-Aparicio, I.Fonseca, J.Bellanato (1992) *J.Mol.Struct.* ,**267,**79

Formula: $C_{20}\ H_{28}\ N_2\ O_6,5(H_2\ O_1)$

Compound Name: 3α-Methoxycarbonyl-3β-(3',4',5'-trimethoxybenzamido)tropane pentahydrate

Space Group:	P-1	***Cell:***	***a***	16.855(2)	***b***	14.181(2)	***c***	10.856(2)
Space Group No.:	2	***(Å,°)***	α	103.28(0)	β	99.17(0)	γ	113.92(0)

R-Factor (%): 4.60 ***Temperature(K):*** 295 ***Density(g/cm³):*** 1.448

Parameters

Fragment 1

**TOR3**	**(T)**	-10.977
**TOR4**	**(T)**	85.893
**TOR5**	**(T)**	-6.440
**C6N2**	**(T)**	-24.144
**C2N2**	**(T)**	154.931

Fragment 2

**TOR3**	**(T)**	-8.299
**TOR4**	**(T)**	-105.309
**TOR5**	**(T)**	-11.669
**C6N2**	**(T)**	145.476
**C2N2**	**(T)**	-38.577

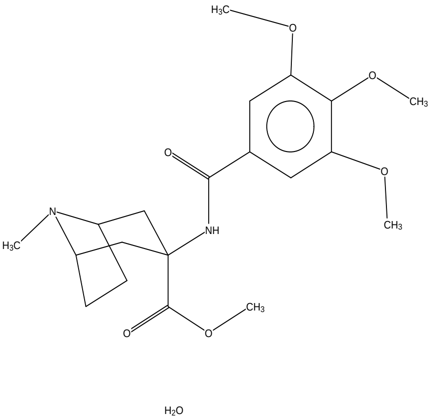

LIRHUQ

Reference: L.K.Charkoudian, D.M.Pham, A.M.Kwon, A.D.Vangeloff, K.J.Franz (2007) *Dalton Trans.* ,5031

Formula: $C_{17}\ H_{18}\ N_2\ O_5$

Compound Name: Salicylaldehyde 3,4,5-trimethoxybenzoyl hydrazone

Space Group:	P21/c	***Cell:***	***a***	14.787(7)	***b***	13.198(6)	***c***	8.225(2)
Space Group No.:	14	***(Å,°)***	α	90.00	β	101.26(0)	γ	90.00

R-Factor (%): 5.00 ***Temperature(K):*** 173 ***Density(g/cm³):*** 1.394

Parameters

Fragment 1

**TOR3**	**(T)**	-14.910
**TOR4**	**(T)**	81.204
**TOR5**	**(T)**	1.869
**C6N2**	**(T)**	142.242
**C2N2**	**(T)**	-36.682

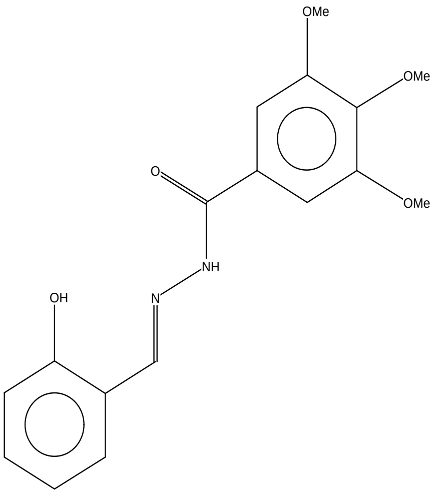

Search: search3 (Wed Mar 16 10:48:50 2016): Hits 13-16

LIRHUQ01

Reference: Yu-Min Wang, Zhen-Dong Zhao, Yu-Xiang Chen, Liang-Wu Bi (2008) *Acta Crystallogr.,Sect.E:Struct.Rep.Online* ,**64,** o1009

Formula: $C_{17} H_{18} N_2 O_5$

Compound Name: (E)-N'-(2-Hydroxybenzylidene)-3,4,5-trimethoxybenzohydrazide

Space Group:	P21/c	***Cell:***	***a*** 15.348(12)	***b*** 13.330(11)	***c*** 8.299(7)		
Space Group No.:	14	***(Å,°)***	α 90.00	β 99.85(1)	γ 90.00		
R-Factor (%):	6.84	***Temperature(K):***	273	***Density(g/cm³):***	1.312		

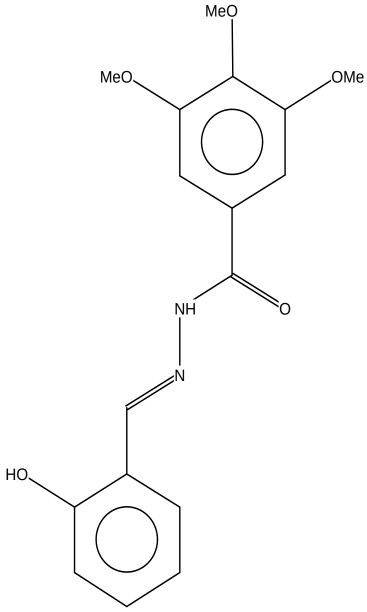

Parameters

Fragment 1

**TOR3**	**(T)**	-18.896
**TOR4**	**(T)**	81.254
**TOR5**	**(T)**	3.005
**C6N2**	**(T)**	141.932
**C2N2**	**(T)**	-36.654

MODSOO

Reference: A.Saeed, R.A.Khera, M.Batool, U.Shaheen, U.Florke (2008) *Acta Crystallogr.,Sect.E:Struct.Rep.Online* ,**64,**o1625

Formula: $C_{16} H_{16} Cl_1 N_1 O_4$

Compound Name: N-(4-Chlorophenyl)-3,4,5-trimethoxybenzamide

Space Group:	Cc	***Cell:***	***a*** 9.487(2)	***b*** 25.666(6)	***c*** 6.978(1)
Space Group No.:	9	***(Å,°)***	α 90.00	β 112.34(0)	γ 90.00
R-Factor (%):	4.50	***Temperature(K):***	120	***Density(g/cm³):***	1.360

Parameters

Fragment 1

**TOR3**	**(T)**	2.909
**TOR4**	**(T)**	-90.826
**TOR5**	**(T)**	19.909
**C6N2**	**(T)**	-31.101
**C2N2**	**(T)**	150.813

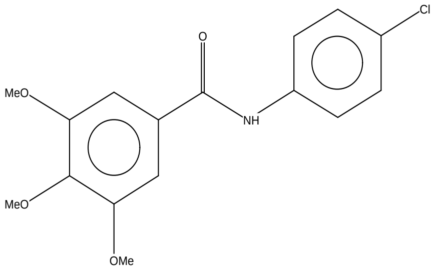

MODTEF

Reference: Yong-Chuang Zhu, Dao-Hang He (2008) *Acta Crystallogr.,Sect.E:Struct.Rep.Online* ,**64,**o1630

Formula: $C_{17} H_{17} Br_1 N_2 O_4,C_1 H_4 O_1$

Compound Name: N'-(2-Bromobenzylidene)-3,4,5-trimethoxybenzohydrazide methanol solvate

Space Group:	Pna21	***Cell:***	***a*** 12.923(0)	***b*** 4.916(0)	***c*** 29.398(1)
Space Group No.:	33	***(Å,°)***	α 90.00	β 90.00	γ 90.00
R-Factor (%):	2.95	***Temperature(K):***	173	***Density(g/cm³):***	1.512

Parameters

Fragment 1

**TOR3**	**(T)**	4.663
**TOR4**	**(T)**	67.757
**TOR5**	**(T)**	2.798
**C6N2**	**(T)**	22.560
**C2N2**	**(T)**	-158.939

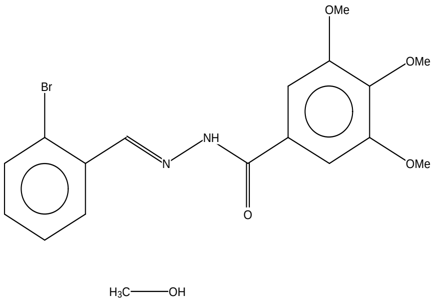

MODTOP

Reference: Hai-Tang Du, Hai-Jun Du (2008) *Acta Crystallogr.,Sect.E:Struct.Rep.Online* ,**64,**o1632

Formula: $C_{28} H_{30} N_4 O_8 S_2,C_2 H_6 O_1$

Compound Name: 3,3'-bis(3,4,5-trimethoxybenzoyl)-1,1'-(o-phenylene)dithiourea ethanol solvate

Space Group:	P-1	***Cell:***	***a*** 7.762(1)	***b*** 14.473(3)	***c*** 15.810(3)
Space Group No.:	2	***(Å,°)***	α 67.11(1)	β 73.07(0)	γ 78.21(1)
R-Factor (%):	4.99	***Temperature(K):***	113	***Density(g/cm³):***	1.409

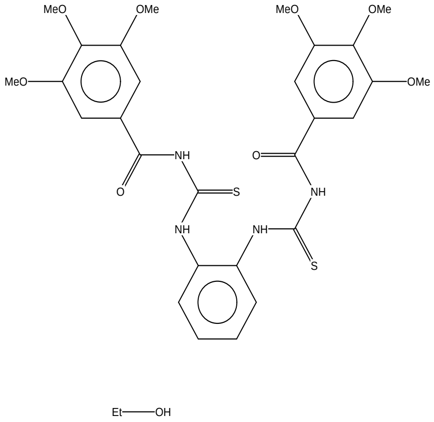

Parameters

Fragment 1

**TOR3**	**(T)**	-5.083
**TOR4**	**(T)**	-92.017
**TOR5**	**(T)**	-2.102
**C6N2**	**(T)**	-162.037
**C2N2**	**(T)**	21.657

Fragment 2

**TOR3**	**(T)**	3.913
**TOR4**	**(T)**	-118.275
**TOR5**	**(T)**	-5.414
**C6N2**	**(T)**	149.992
**C2N2**	**(T)**	-31.787

Search: search3 (Wed Mar 16 10:48:50 2016): Hits 17-20

MODXAF

Reference: Dao-Hang He, Yong-Chuang Zhu, Zhuo-Ru Yang (2008)
Acta Crystallogr.,Sect.E:Struct.Rep.Online ,**64,**o1648

Formula: C_{17} H_{17} F_1 N_2 O_4

Compound Name: N'-(4-Fluorobenzylidene)-3,4,5-trimethoxybenzohydrazide

Space Group: P21/c
Space Group No.: 14

Cell: ***(Å,°)*** ***a*** 7.919(0) ***b*** 26.250(1) ***c*** 8.127(0) α 90.00 β 105.55(0) γ 90.00

R-Factor (%): 3.70 ***Temperature(K):*** 173 ***Density(g/cm³):*** 1.356

Parameters

Fragment 1

**TOR3 (T)**	-19.343
**TOR4 (T)**	97.393
**TOR5 (T)**	10.439
**C6N2 (T)**	142.263
**C2N2 (T)**	-35.627

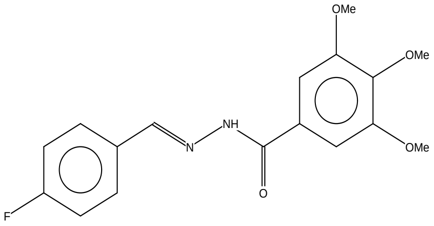

MODXEJ

Reference: Dao-Hang He, Yong-Chuang Zhu, Zhuo-Ru Yang, Shao-Yun Song, Qi-Jin Chen (2008)
Acta Crystallogr.,Sect.E:Struct.Rep.Online ,**64,**o1649

Formula: C_{17} H_{17} Cl_1 N_2 O_4,C_1 H_4 O_1

Compound Name: N'-(2-Chlorobenzylidene)-3,4,5-trimethoxybenzohydrazide methanol solvate

Space Group: Pna21
Space Group No.: 33

Cell: ***(Å,°)*** ***a*** 12.936(0) ***b*** 4.872(0) ***c*** 29.412(1) α 90.00 β 90.00 γ 90.00

R-Factor (%): 3.13 ***Temperature(K):*** 173 ***Density(g/cm³):*** 1.365

Parameters

Fragment 1

**TOR3 (T)**	-4.486
**TOR4 (T)**	-66.323
**TOR5 (T)**	-2.297
**C6N2 (T)**	-23.067
**C2N2 (T)**	158.861

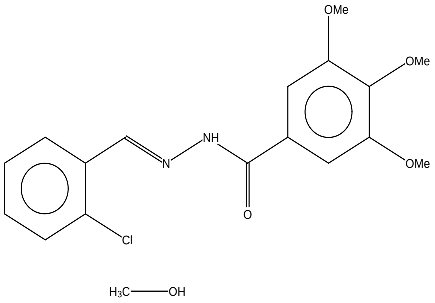

NICHOY

Reference: A.Saeed, J.Simpson (2013) *J.Chem.Cryst.* ,**43,**51

Formula: C_{17} H_{19} N_1 O_4

Compound Name: 3,4,5-Trimethoxy-N-(4-methylphenyl)benzamide

Synonym: 3,4,5-trimethoxy-N-p-tolylbenzamide

Space Group: P21
Space Group No.: 4

Cell: ***(Å,°)*** ***a*** 5.107(0) ***b*** 13.915(1) ***c*** 11.205(1) α 90.00 β 103.12(0) γ 90.00

R-Factor (%): 3.77 ***Temperature(K):*** 90 ***Density(g/cm³):*** 1.291

Parameters

Fragment 1

**TOR3 (T)**	-1.385
**TOR4 (T)**	-108.897
**TOR5 (T)**	-1.858
**C6N2 (T)**	148.362
**C2N2 (T)**	-34.473

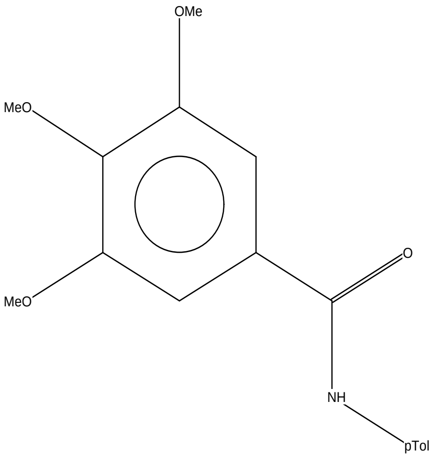

NIQPEJ

Reference: Hai-Tang Du, Hai-Jun Du, Ming Lu, Li-Li Sun (2007)
Acta Crystallogr.,Sect.E:Struct.Rep.Online ,**63,**o4827

Formula: C_{19} H_{22} N_2 O_4 S_1

Compound Name: 1-(2,6-Dimethylphenyl)-3-(3,4,5-trimethoxybenzoyl)thiourea

Space Group: P21/c
Space Group No.: 14

Cell: ***(Å,°)*** ***a*** 11.610(3) ***b*** 7.456(1) ***c*** 22.085(5) α 90.00 β 102.75(0) γ 90.00

R-Factor (%): 3.96 ***Temperature(K):*** 294 ***Density(g/cm³):*** 1.334

Parameters

Fragment 1

**TOR3 (T)**	-2.080
**TOR4 (T)**	-95.960
**TOR5 (T)**	10.866
**C6N2 (T)**	-18.808
**C2N2 (T)**	166.429

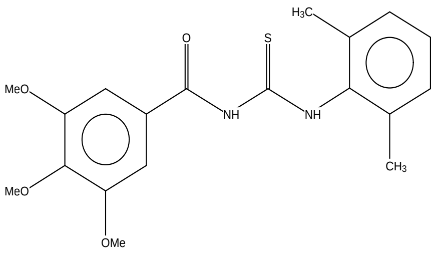

Search: search3 (Wed Mar 16 10:48:50 2016): Hits 21-24

NUQJUF

Reference: A.Saeed, U.Shaheen, U.Florke (2010) *Acta Crystallogr.,Sect.E:Struct.Rep.Online* ,**66,**o1133

Formula: C_{17} H_{17} F_1 N_2 O_4 S_1

Compound Name: 1-(2-Fluorophenyl)-3-(3,4,5-trimethoxybenzoyl)thiourea

Synonym: N-((2-Fluorophenyl)carbamothioyl)-3,4,5-trimethoxybenzamide

Space Group: P-1
Space Group No.: 2
Cell: (Å,°) ***a*** 4.083(0) ***b*** 14.042(1) ***c*** 14.229(1); α 91.09(0) β 90.69(0) γ 91.71(0)

R-Factor (%): 4.23 ***Temperature(K):*** 120 ***Density(g/cm3):*** 1.484

Parameters

Fragment 1

**TOR3 (T)**	-5.830
**TOR4 (T)**	102.966
**TOR5 (T)**	-5.868
**C6N2 (T)**	-160.204
**C2N2 (T)**	26.091

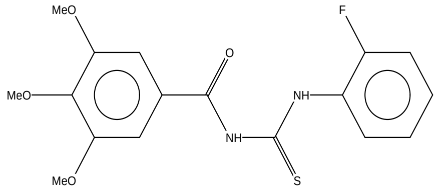

NUQLIV

Reference: H.Choi, B.H.Han, T.Lee, S.K.Kang, C.K.Sung (2010) *Acta Crystallogr.,Sect.E:Struct.Rep.Online* ,**66,**o1142

Formula: C_{16} H_{15} F_2 N_1 O_4

Compound Name: N-(3,4-Difluorophenyl)-3,4,5-trimethoxybenzamide

Space Group: P21/n
Space Group No.: 14
Cell: (Å,°) ***a*** 5.003(0) ***b*** 8.899(0) ***c*** 32.726(2); α 90.00 β 93.90(0) γ 90.00

R-Factor (%): 6.51 ***Temperature(K):*** 174 ***Density(g/cm3):*** 1.477

Parameters

Fragment 1

**TOR3 (T)**	5.320
**TOR4 (T)**	-70.389
**TOR5 (T)**	3.441
**C6N2 (T)**	32.870
**C2N2 (T)**	-150.304

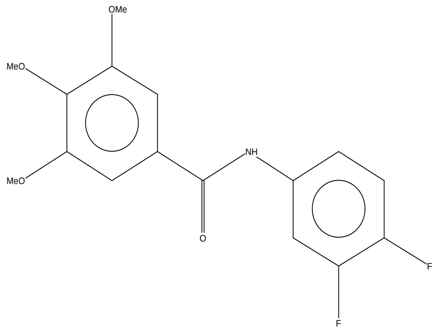

PAVMAC

Reference: Wen Gu, Chao Qiao (2012) *Acta Crystallogr.,Sect.E:Struct.Rep.Online* ,**68,**o1658

Formula: C_{16} H_{16} Br_1 N_1 O_4

Compound Name: N-(4-Bromophenyl)-3,4,5-trimethoxybenzamide

Space Group: Cc
Space Group No.: 9
Cell: (Å,°) ***a*** 9.586(1) ***b*** 26.010(5) ***c*** 7.139(1); α 90.00 β 112.04(3) γ 90.00

R-Factor (%): 4.47 ***Temperature(K):*** 293 ***Density(g/cm3):*** 1.474

Parameters

Fragment 1

**TOR3 (T)**	-20.854
**TOR4 (T)**	-91.180
**TOR5 (T)**	-4.560
**C6N2 (T)**	-146.396
**C2N2 (T)**	36.434

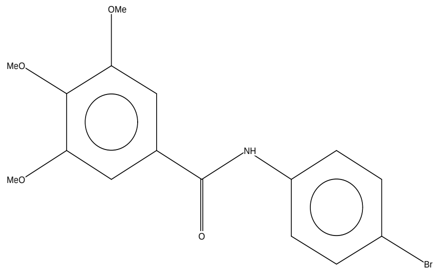

PIDTEC

Reference: Yi-Feng Sun, Xian-Zhong Sun, Ji-Kun Li, Ze-Bao Zheng (2007) *Acta Crystallogr.,Sect.E:Struct.Rep.Online* ,**63,**o2180

Formula: C_{19} H_{22} N_2 O_7,H_2 O_1

Compound Name: 4-Hydroxy-3,5-dimethoxybenzaldehyde 3,4,5-trimethoxybenzoylhydrazone monohydrate

Space Group: P21/c
Space Group No.: 14
Cell: (Å,°) ***a*** 10.130(0) ***b*** 13.811(0) ***c*** 14.876(0); α 90.00 β 109.60(0) γ 90.00

R-Factor (%): 5.33 ***Temperature(K):*** 273 ***Density(g/cm3):*** 1.384

Parameters

Fragment 1

**TOR3 (T)**	15.016
**TOR4 (T)**	-98.640
**TOR5 (T)**	-85.859
**C6N2 (T)**	164.182
**C2N2 (T)**	-15.396

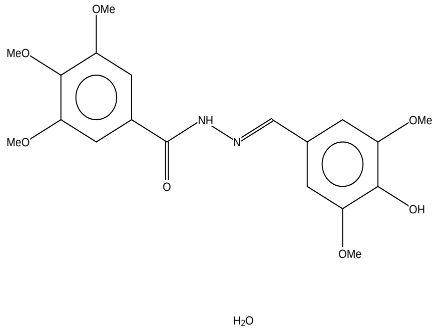

Search: search3 (Wed Mar 16 10:48:50 2016): Hits 25-28

PIXJIQ

Reference: L.Bonardi, H.Kanaan, F.Camerel, P.Jolinat, P.Retailleau, R.Ziessel (2008) *Adv.Funct.Mater.* ,**18,**401

Formula: $C_{33}\ H_{38}\ B_1\ F_2\ N_3\ O_4$

Compound Name: 2,6-Diethyl-4,4-difluoro-1,3,5,7-tetramethyl-8-(4-(3,4,5-trimethoxybenzoylamino)phenyl)-4,4a-dihydro-3a,4a-diaza-4-bora-s-indacene

Space Group:	P21/c	***Cell:***	***a*** 15.036(1)	***b*** 21.640(2)	***c*** 10.101(1)
Space Group No.:	14	***(Å,°)***	α 90.00	β 106.84(0)	γ 90.00
R-Factor (%):	5.24	***Temperature(K):*** 293	***Density(g/cm³):*** 1.245		

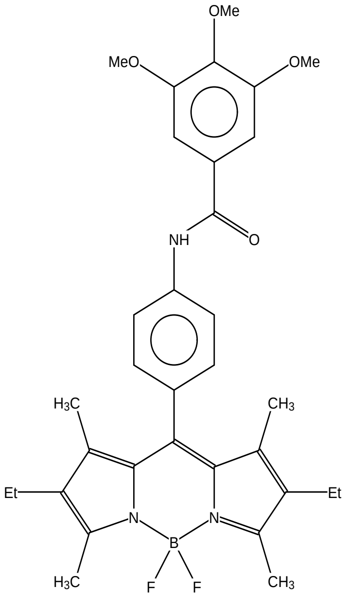

Parameters

Fragment 1

**TOR3 (T)**	4.546
**TOR4 (T)**	86.916
**TOR5 (T)**	7.302
**C6N2 (T)**	-29.729
**C2N2 (T)**	148.584

QOCDET

Reference: Y.Ishikawa, K.Watanabe (2014) *Acta Crystallogr.,Sect.E:Struct.Rep.Online* ,**70,**o472

Formula: $C_{20}\ H_{18}\ N_2\ O_6$

Compound Name: 3,4,5-trimethoxy-N'-((4-oxo-4H-chromen-3-yl)methylene) benzohydrazide

Space Group:	P-1	***Cell:***	***a*** 6.628(7)	***b*** 11.029(18)	***c*** 12.544(18)
Space Group No.:	2	***(Å,°)***	α 105.34(12)	β 96.94(10)	γ 95.88(10)
R-Factor (%):	6.84	***Temperature(K):*** 100	***Density(g/cm³):*** 1.461		

Parameters

Fragment 1

**TOR3 (T)**	-0.771
**TOR4 (T)**	80.845
**TOR5 (T)**	-22.614
**C6N2 (T)**	-152.511
**C2N2 (T)**	29.977

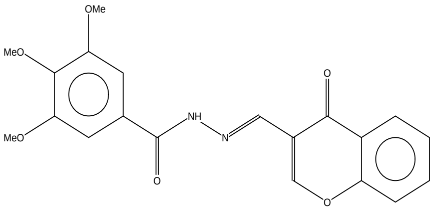

RIGCOA

Reference: M.Zareef, R.Iqbal, G.Qadeer, M.Arfan, Wai-Yeung Wong (2007) *Acta Crystallogr.,Sect.E:Struct.Rep.Online* ,**63,**o3052

Formula: $C_{17}\ H_{17}\ F_1\ N_2\ O_5{,}H_2\ O_1$

Compound Name: N'-(4-Fluorobenzoyl)-3,4,5-trimethoxybenzohydrazide monohydrate

Space Group:	P21/c	***Cell:***	***a*** 8.385(0)	***b*** 13.269(0)	***c*** 31.833(2)
Space Group No.:	14	***(Å,°)***	α 90.00	β 94.62(0)	γ 90.00
R-Factor (%):	4.81	***Temperature(K):*** 294	***Density(g/cm³):*** 1.379		

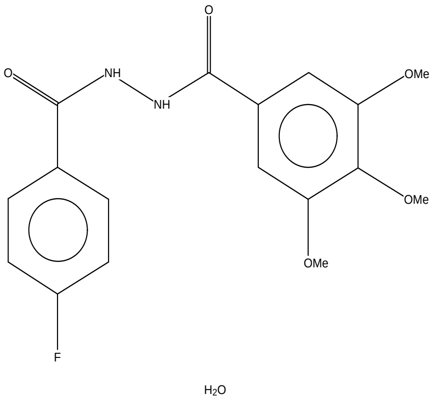

Parameters

Fragment 1

**TOR3 (T)**	-20.599
**TOR4 (T)**	78.960
**TOR5 (T)**	1.772
**C6N2 (T)**	30.758
**C2N2 (T)**	-152.478

Fragment 2

**TOR3 (T)**	23.294
**TOR4 (T)**	-80.233
**TOR5 (T)**	9.267
**C6N2 (T)**	-34.219
**C2N2 (T)**	150.568

SUJFUA

Reference: T.K.Achar, P.Mal (2014) *J.Org.Chem.* ,**80,**666

Formula: $C_{17}\ H_{19}\ N_1\ O_4$

Compound Name: N-Benzyl-3,4,5-trimethoxybenzamide

Space Group:	P21/c	***Cell:***	***a*** 23.000(0)	***b*** 5.091(0)	***c*** 12.942(0)
Space Group No.:	14	***(Å,°)***	α 90.00	β 95.10(0)	γ 90.00
R-Factor (%):	3.99	***Temperature(K):*** 298	***Density(g/cm³):*** 1.326		

Parameters

Fragment 1

**TOR3 (T)**	1.670
**TOR4 (T)**	76.043
**TOR5 (T)**	3.079
**C6N2 (T)**	-41.227
**C2N2 (T)**	144.461

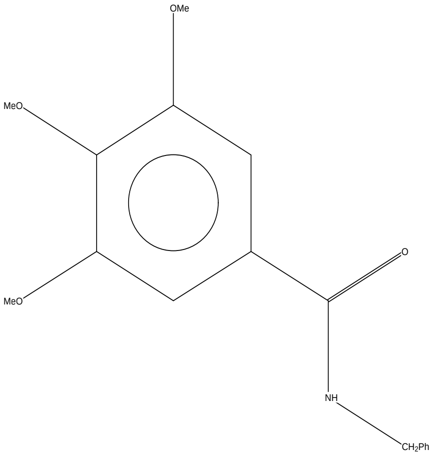

Search: search3 (Wed Mar 16 10:48:50 2016): Hits 29-32

TIQCON

Reference: C.C.Paraschivescu, M.Matache, C.Dobrota, A.Nicolescu, C.Maxim, C.Deleanu, I.C.Farcasanu, N.D.Hadade (2013) *J.Org.Chem.* ,**78,**2670

Formula: $C_{28}\,H_{30}\,N_4\,O_8$

Compound Name: 3,4,5-Trimethoxy-N-(1-((3,4,5-trimethoxybenzoyl)hydrazono)-1,3-dihydro-2H-isoindol-2-yl)benzamide

Space Group:	P21/a	***Cell:***	***a*** 10.204(1)	***b*** 14.265(1)	***c*** 18.965(3)
Space Group No.:	14	***(Å,°)***	α 90.00	β 97.12(1)	γ 90.00

R-Factor (%): 7.78 ***Temperature(K):*** 293 ***Density(g/cm^3):*** 1.335

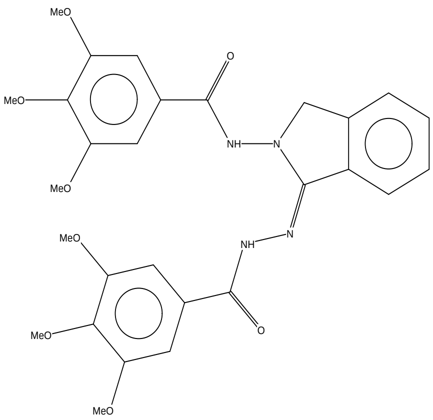

Parameters

Fragment 1

**TOR3 (T)**	-7.866
**TOR4 (T)**	-114.015
**TOR5 (T)**	11.233
**C6N2 (T)**	164.497
**C2N2 (T)**	-14.908

Fragment 2

**TOR3 (T)**	0.966
**TOR4 (T)**	-100.904
**TOR5 (T)**	-3.280
**C6N2 (T)**	-41.150
**C2N2 (T)**	140.508

VELYIV

Reference: M.Zareef, R.Iqbal, G.Qadeer, M.Arfan, Xiaoming Lu (2006) *Acta Crystallogr.,Sect.E:Struct.Rep.Online* ,**62,**o3259

Formula: $C_{10}\,H_{14}\,N_2\,O_4$,0.5($H_2\,O_1$)

Compound Name: 3,4,5-Trimethoxybenzohydrazide hemihydrate

Space Group:	C2/c	***Cell:***	***a*** 33.253(6)	***b*** 4.836(0)	***c*** 14.227(3)
Space Group No.:	15	***(Å,°)***	α 90.00	β 93.07(1)	γ 90.00

R-Factor (%): 4.77 ***Temperature(K):*** 273 ***Density(g/cm^3):*** 1.368

Parameters

Fragment 1

**TOR3 (T)**	-7.532
**TOR4 (T)**	-79.416
**TOR5 (T)**	-2.170
**C6N2 (T)**	-171.459
**C2N2 (T)**	8.302

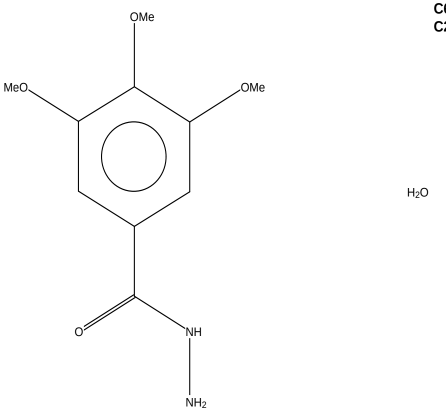

VOJJOU

Reference: Zhen-dong Zhao, Yu-min Wang, Yu-xiang Chen, Liang-wu Bi (2008) *Acta Crystallogr.,Sect.E:Struct.Rep.Online* ,**64,** o2408

Formula: $C_{17}\,H_{17}\,Br_1\,N_2\,O_5$

Compound Name: (E)-N'-(5-bromo-2-hydroxybenzylidene)-3,4,5-trimethoxybenzohydrazide

Space Group:	P21/c	***Cell:***	***a*** 11.416(1)	***b*** 16.279(3)	***c*** 9.374(1)
Space Group No.:	14	***(Å,°)***	α 90.00	β 100.21(0)	γ 90.00

R-Factor (%): 3.69 ***Temperature(K):*** 273 ***Density(g/cm^3):*** 1.586

Parameters

Fragment 1

**TOR3 (T)**	5.454
**TOR4 (T)**	116.220
**TOR5 (T)**	-6.453
**C6N2 (T)**	147.850
**C2N2 (T)**	-33.045

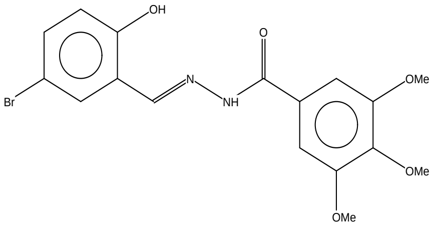

VOJKEL

Reference: Yu-Min Wang, Zhen-Dong Zhao, Yu-Xiang Chen, Liang-Wu Bi (2008) *Acta Crystallogr.,Sect.E:Struct.Rep.Online* ,**64,** o2459

Formula: $C_{17}\,H_{17}\,Cl_1\,N_2\,O_4$

Compound Name: (E)-N'-(4-chlorobenzylidene)-3,4,5-trimethoxybenzohydrazide

Space Group:	P-1	***Cell:***	***a*** 5.119(2)	***b*** 8.210(4)	***c*** 20.276(9)
Space Group No.:	2	***(Å,°)***	α 101.06(0)	β 92.36(0)	γ 101.46(0)

R-Factor (%): 4.61 ***Temperature(K):*** 273 ***Density(g/cm^3):*** 1.418

Parameters

Fragment 1

**TOR3 (T)**	-9.046
**TOR4 (T)**	-63.651
**TOR5 (T)**	3.413
**C6N2 (T)**	-145.945
**C2N2 (T)**	37.920

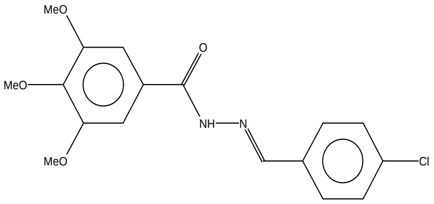

Search: search3 (Wed Mar 16 10:48:50 2016): Hits 33-36

WOMRIA

Reference: D.Uraguchi, Y.Ueki, T.Ooi (2008) *J.Am.Chem.Soc.* ,**130,** 14088

Formula: $C_{36} H_{40} N_2 O_8 S_1,C_4 H_8 O_2$

Compound Name: α-(1-(((2,5-Dimethylphenyl)sulfonyl)amino)-3-phenylpropyl)-N-(3,4,5-trimethoxybenzoyl)phenylalanine ethyl acetate solvate

Space Group:	P21	***Cell:***	***a***	11.360(3)	***b***	50.827(12)	***c***	13.745(3)
Space Group No.:	4	***(Å,°)***	α	90.00	β	90.21(0)	γ	90.00

R-Factor (%): 6.53 ***Temperature(K):*** 153 ***Density(g/cm³):*** 1.254

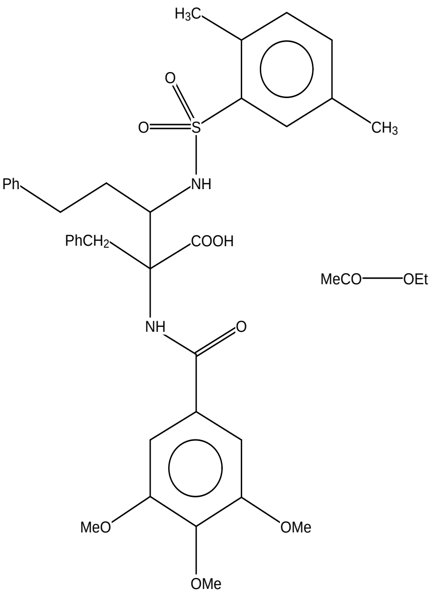

Parameters

Fragment 1	
**TOR3 (T)**	4.384
**TOR4 (T)**	76.166
**TOR5 (T)**	-3.081
**C6N2 (T)**	-151.243
**C2N2 (T)**	28.170

Fragment 2	
**TOR3 (T)**	2.315
**TOR4 (T)**	-95.585
**TOR5 (T)**	-4.412
**C6N2 (T)**	137.073
**C2N2 (T)**	-44.215

Fragment 3	
**TOR3 (T)**	1.644
**TOR4 (T)**	-95.057
**TOR5 (T)**	-4.521
**C6N2 (T)**	136.677
**C2N2 (T)**	-43.884

Fragment 4	
**TOR3 (T)**	-3.556
**TOR4 (T)**	-108.774
**TOR5 (T)**	5.064
**C6N2 (T)**	26.545
**C2N2 (T)**	-151.318

YAGBIT

Reference: Hyeong Choi, Yong Suk Shim, Byung Hee Han, Sung Kwon Kang, Chang Keun Sung (2011) *Acta Crystallogr.,Sect.E:Struct.Rep.Online* ,**67,**o2865

Formula: $C_{16} H_{17} N_1 O_5$

Compound Name: N-(4-Hydroxyphenyl)-3,4,5-trimethoxybenzamide

Space Group:	Pbca	***Cell:***	***a***	10.428(0)	***b***	13.408(0)	***c***	21.556(0)
Space Group No.:	61	***(Å,°)***	α	90.00	β	90.00	γ	90.00

R-Factor (%): 5.42 ***Temperature(K):*** 296 ***Density(g/cm³):*** 1.337

Parameters

Fragment 1	
**TOR3 (T)**	6.642
**TOR4 (T)**	44.218
**TOR5 (T)**	4.066
**C6N2 (T)**	151.333
**C2N2 (T)**	-32.384

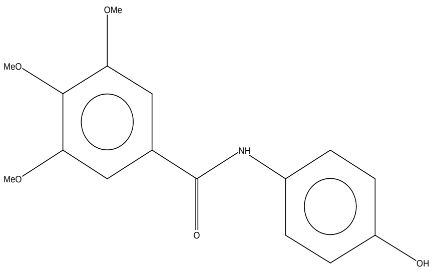

YOZRAH

Reference: R.J.Warr, A.N.Westra, K.J.Bell, J.Chartres, R.Ellis, C.Tong, T.G.Simmance, A.Gadzhieva, A.J.Blake, P.A.Tasker, M.Schroder (2009) *Chem.-Eur.J.* ,**15,**4386

Formula: $C_{36} H_{48} N_4 O_{12},C_1 H_2 Cl_2,H_2 O_1$

Compound Name: tris(2-(N-(3,4,5-Trimethoxybenzoyl)amino)ethyl)amine dichloromethane solvate monohydrate

Space Group:	P-1	***Cell:***	***a***	10.103(0)	***b***	12.325(0)	***c***	17.639(1)
Space Group No.:	2	***(Å,°)***	α	73.01(0)	β	88.58(0)	γ	77.19(0)

R-Factor (%): 4.21 ***Temperature(K):*** 150 ***Density(g/cm³):*** 1.350

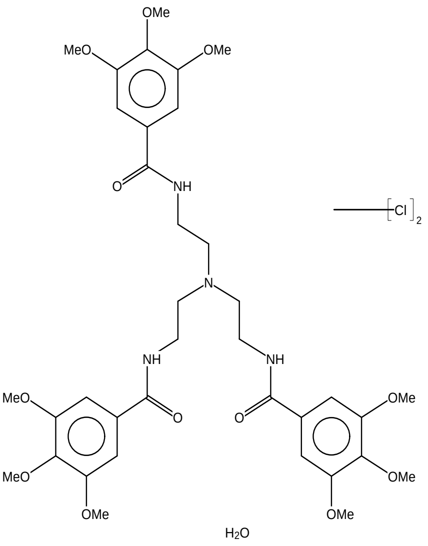

Parameters

Fragment 1	
**TOR3 (T)**	7.691
**TOR4 (T)**	116.549
**TOR5 (T)**	-17.323
**C6N2 (T)**	160.497
**C2N2 (T)**	-20.822

Fragment 2	
**TOR3 (T)**	-4.654
**TOR4 (T)**	116.555
**TOR5 (T)**	7.158
**C6N2 (T)**	15.611
**C2N2 (T)**	-166.466

Fragment 3	
**TOR3 (T)**	3.304
**TOR4 (T)**	-97.083
**TOR5 (T)**	-8.952
**C6N2 (T)**	-16.452
**C2N2 (T)**	163.607

JACHUT

Reference: You-Yue Han (2013) *J.Chil.Chem.Soc.* ,**58,**1858

Formula: $C_{17} H_{16} Cl_2 N_2 O_4$

Compound Name: N'-(2,4-dichlorobenzylidene)-3,4,5-trimethoxybenzohydrazide

Space Group:	P21/c	***Cell:***	***a***	17.127(3)	***b***	13.326(3)	***c***	7.984(1)
Space Group No.:	14	***(Å,°)***	α	90.00	β	99.69(0)	γ	90.00

R-Factor (%): 4.89 ***Temperature(K):*** 298 ***Density(g/cm³):*** 1.417

Parameters

Fragment 1	
**TOR3 (T)**	0.343
**TOR4 (T)**	-102.909
**TOR5 (T)**	-19.493
**C6N2 (T)**	-38.862
**C2N2 (T)**	138.900

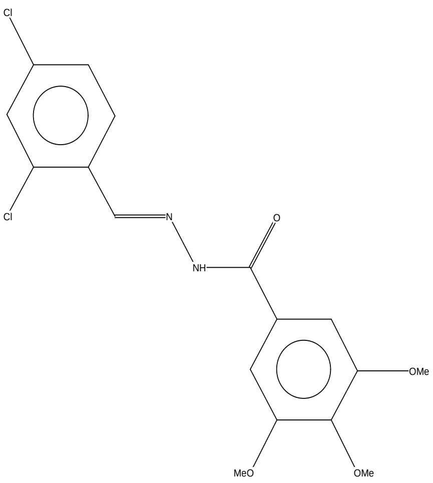

Search: search3 (Wed Mar 16 10:48:50 2016): Hit 37

JACJAB

Reference: You-Yue Han (2013) *J.Chil.Chem.Soc.* ,**58,**1858

Formula: $C_{17} H_{18} N_2 O_6$

Compound Name: N'-(2,5-dihydroxybenzylidene)-3,4,5-trimethoxybenzohydrazide

Space Group:	Pbca	***Cell:***	***a***	15.193(2)	***b***	11.087(1)	***c***	20.203(3)
Space Group No.:	61	***(Å,°)***	α	90.00	β	90.00	γ	90.00

R-Factor (%): 4.53 ***Temperature(K):*** 298 ***Density(g/cm^3):*** 1.352

Parameters

Fragment 1

**TOR3**	**(T)**	-8.576
**TOR4**	**(T)**	-101.844
**TOR5**	**(T)**	5.290
**C6N2**	**(T)**	30.985
**C2N2**	**(T)**	-147.421

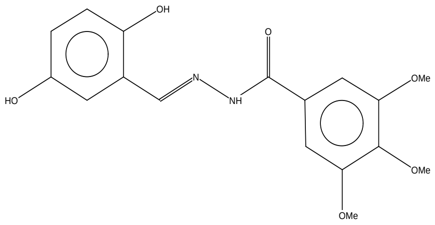

Supplement: Supplementary file 9 [file e-72-00675-sup9.pdf]
